# A Quarter Century of EHD Protein Research: From Endosomal Recycling to Ciliopathies

**DOI:** 10.1111/tra.70039

**Published:** 2026-06-20

**Authors:** Devin Frisby, Gunjan Misri, Naava Naslavsky, Steve Caplan

**Affiliations:** ^1^ Department of Biochemistry and Molecular Biology University of Nebraska Medical Center Omaha Nebraska USA; ^2^ Fred and Pamela Buffett Cancer Center, University of Nebraska Medical Center Omaha Nebraska USA

**Keywords:** ATPase, ciliogenesis, EHD1, EHD2, EHD3, EHD4, endosome fission, membrane remodelling, membrane trafficking, receptor recycling

## Abstract

Eps15 homology domain‐containing proteins comprise a conserved family of membrane‐remodeling ATPases that regulate endocytic trafficking, membrane fission, receptor recycling, primary ciliogenesis and membrane dynamics across eukaryotes. Since the initial identification of EHD1 and its *Caenorhabditis elegans* homolog RME‐1 as regulators of endocytic recycling, research over the past quarter century has expanded the functional scope of EHD proteins far beyond classical receptor return to the plasma membrane. In mammals, EHD1, EHD2, EHD3, and EHD4 occupy overlapping but distinct cellular locations and regulate diverse processes including tubular recycling endosome fission, caveolae stabilization, primary ciliogenesis, centrosome duplication, cytokinesis, mitochondrial homeostasis, lipid droplet biology, and lipophagy. These cellular functions are supported by extensive studies in cultured cells and animal models, including mice, zebrafish, flies, worms, and plants, highlighting both conserved and specialized roles for EHD orthologs. EHD dysfunction has also been associated with a broad range of human diseases, including metabolic and cardiovascular disorders, inflammatory and infectious disease, neurologic conditions, cancer, and ciliopathies. Although many disease links remain correlative or model‐based, the recent identification of an EHD1 founder mutation causing proteinuria, hearing loss, and polycystic kidney disease provides direct genetic evidence connecting EHD dysfunction to human pathology. This review summarizes 25 years of EHD research, emphasizing how EHD proteins coordinate membrane trafficking, organelle remodeling, and disease‐relevant cellular physiology.

## Graphical abstract

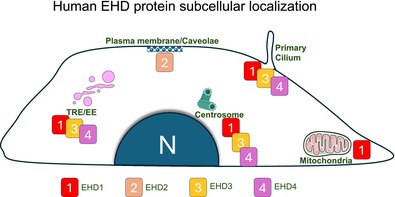

**Abbreviations**
BBS1: Bardet‐Biedl Syndrome 1
EHD: Eps15 Homology Domain
ERC: endocytic recycling compartment
MHC‐I: Major Histocompatibility Class 1
MICAL‐L1: MICAL‐like protein 1
PM: plasma membrane
RME‐1: receptor‐mediated endocytosis‐1
TRE: tubular recycling endosomes

## 1 Background and History

The Eps15 Homology Domain‐containing protein EHD1 was first identified as the product of a highly conserved gene present in invertebrates such as *Drosophila melanogaster* and *Caenorhabditis elegans*, as well as mammals including mice and humans. Based on its sequence, domain architecture, and predicted interaction partners, it was proposed to play a role in endocytosis [1]. Owing to its chromosomal location, EHD1 was initially proposed as a candidate gene for Bardet‐Biedl syndrome (BBS), reflecting early interest in its potential disease relevance [2]. Almost two decades later, it was demonstrated that EHD1 regulates primary ciliogenesis [3, 4]. Remarkably, nearly a quarter century after its discovery, a point mutation in EHD1 was directly linked to human disease, causing tubular proteinuria and hearing loss, consistent with a ciliopathy [5].

The primary function attributed to EHD1 and its *C. elegans* homolog RME‐1 in the early 2000s was the regulation of endocytic trafficking, particularly the recycling of internalized receptors back to the plasma membrane (PM) [6, 7, 8]. Over the past 25 years, numerous studies have confirmed that EHD1 controls the recycling of multiple receptors to the cell surface (see Table 1), with knockdown or depletion of EHD1 resulting in delayed recycling and intracellular retention of receptors [59, 60, 61]. While these observations have been repeatedly validated, the mechanistic understanding of how EHD1 regulates endocytic transport has evolved. Accumulating evidence now converges on a central role for EHD1 in mediating endosomal fission. Together, these findings established EHD1 as a central regulator of endocytic trafficking, paving the way for later work implicating it in other cellular processes.

**TABLE 1:** Receptors regulated by EHD proteins.

EHD paralog	Plasma membrane protein	Model	References
EHD1	Yolk receptor	*C. elegans*	[6]
	Transferrin receptor	Chinese Hamster Ovary (CHO) cells, MEFs, HeLa	[7, 9, 10]
	MHC‐I	HeLa cells	[8]
	β1 integrin	HeLa cells	[11]
	CD59	HeLa cells	[12]
	MHC‐II	HeLa cells	[13]
	AMPA‐type glutamate receptors	Primary hippocampal neurons (rats)	[14]
	Cystic fibrosis transmembrane conductance regulator	HEK293‐CFTR	[15]
	Hyperpolarization‐activated cyclic nucleotide‐gated (HCN) ion channels HCN1, HCN2, and HCN4	Opossum kidney cells	[16]
	Potassium channel KCa2.3	HEK293 and HMEC‐1 cells	[17]
	G‐protein‐activated inwardly rectifying potassium channels (GIRK)	Primary hippocampal neurons (rats)	[18]
	LRP1	NIH3T3	[19]
	Smoothened receptor	NIH3T3	[20]
	TGFβR1	HTR8 cells	[21]
	IGF‐1R	TC71 NTC cells	[22]
	EGFR	MEFs, mice mammary epithelial cells	[23]
	Cx43	Cardiomyocytes (mouse)	[24]
	CD44	Lung adenocarcinoma and glioma cells	[25, 26]
	Colony‐Stimulating Factor‐1 (CSF‐1) receptor	Bone marrow–derived macrophages (BMDMs) (mouse)	[27]
	TNFR2	Bone marrow–derived macrophages (BMDMs) (mouse)	[28]
	Neuro‐glia cell adhesion molecule (NgCAM)	Hippocampal neurons (rat)	[29]
	Fer‐1‐like‐5 (Fer1L5)	Myoblasts (mouse)	[30]
	Src *non‐receptor tyrosine kinase	HeLa	[31]
	β2‐adrenergic receptors (β2AR)	Non‐small cell lung cancer cells	[32]
	LDLR	MEFs	[33]
EHD2	LeEix2 receptor	*Nicotiana tabacum cv samsun* and *Nicotiana benthamiana* leaves	[34, 35]
	GLUT4 transporter	3T3‐L1 adipocytes	[36]
	Transferrin receptor	3T3‐L1 adipocytes, *Arabidopsis thaliana*	[36, 37]
	Delta‐like ligand 4 (Dll4)	Human umbilical vein endothelial cell	[38]
	Myoferlin	Myoblasts (mouse)	[39, 40]
	K_ATP_ channel	Ventricular myocytes (mouse)	[41]
	eNOS	HUVECs	[42]
	ORAI1	Triple‐negative breast cancer cells (HS87 and MDA‐MB cells)	[43]
	E‐cadherin	Breast carcinoma, hepatocellular carcinoma, esophageal squamous carcinoma	[44, 45, 46]
EHD3	Transferrin receptor	HeLa cells	[47]
	CD59	HeLa cells	[12]
	CI‐M6PR	HeLa cells	[48]
	CaV3.1	Atrial myocytes (mouse)	[49]
	CaV3.2	Atrial myocytes (mouse)	[49]
	EGFR	U251 cells	[50]
	β3 integrin	HeLa cells	[51]
	Na/Ca exchanger	Cardiac myocytes (mouse)	[52]
	L‐type calcium channel type 1.2 (CaV1.2)	Cardiac myocytes (mouse)	[52]
EHD4	Nogo‐A	Primary hippocampal neurons (rats)	[53]
	TrkA	PC12 cells	[54]
	MHC‐I	HeLa cells	[55]
	Transferrin receptor	HeLa cells	[55]
	LDL receptor	HeLa cells	[55]
	VE‐cadherin	HUVECs	[56]
	Na+ transport channel Na_v_1.5	Ventricular cardiomyocytes (mouse)	[57]
	CD59	HeLa cells	[12]
^—^ ^a^	TCR‐CD3	Primary spleen cells (mice)	[58]

^a^ EHD1, EHD3, and EHD4 triple knockout cells.

In mammals, the EHD protein family (often referred to as the “C‐terminal EHD proteins,” because of the unusual positioning of their Eps15 homology (EH) domain at the carboxyl terminus) comprises four closely related members: EHD1, EHD2, EHD3, and EHD4. In this review, we examine the physiological roles of EHD1 and its paralogs in human disease. At the cellular level, we will highlight their involvement in diverse processes, including receptor and membrane recycling, caveolae stabilization, primary ciliogenesis, centrosome duplication and cell division, and mitochondrial homeostasis. We will also discuss the functions of EHD proteins expressed in animal models including mice, worms, flies, and zebrafish, as well as in plants. Potential mechanisms of EHD activity will be considered, with particular emphasis on structure and binding partners, lipid binding, ATP binding and hydrolysis, and membrane fission. Finally, we outline future directions for EHD research.

## 2 Physiological Functions of EHDs

**FIGURE 1:**
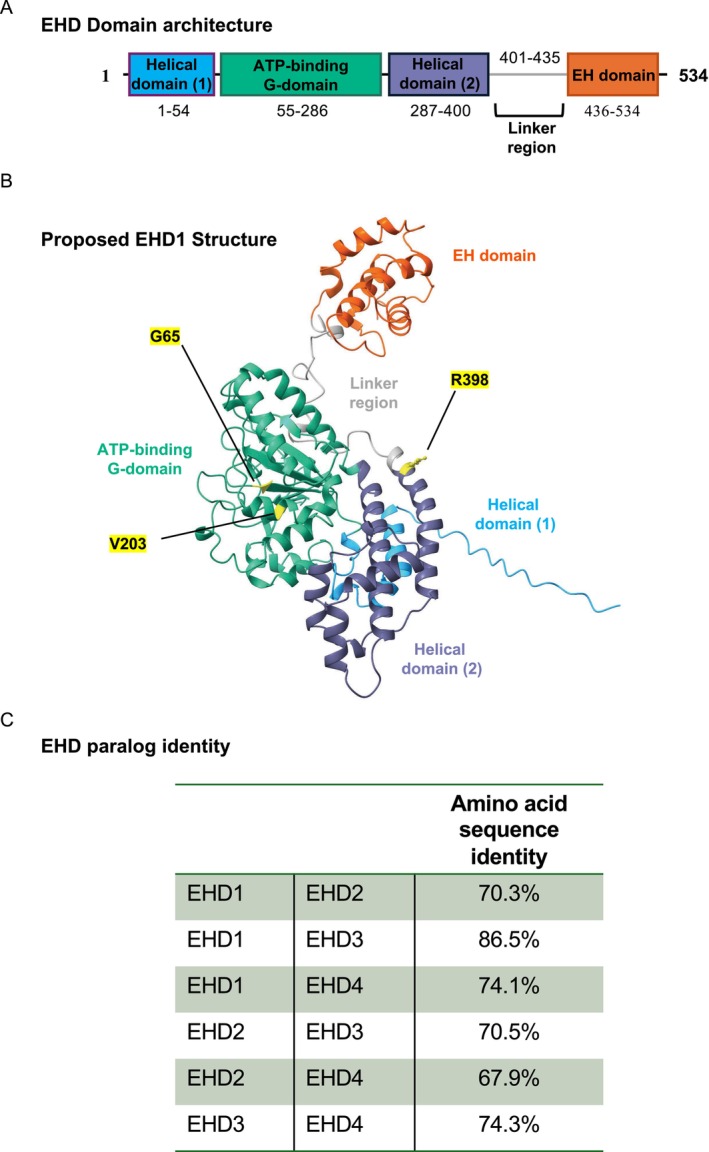
EHD protein domain architecture, structure and homology between human paralogs. (A) Domain architecture of EHD1. (B) Proposed EHD1 structure from UniProt. (C) EHD protein paralog amino acid identity.

**FIGURE 2:**
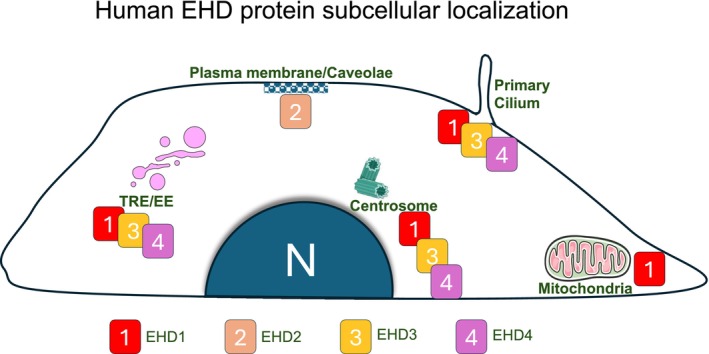
Human EHD protein subcellular localization. EE, early or sorting endosome; TRE, tubular recycling endosome.

The C‐terminal *E*ps15 *h*omology *d*omain‐containing protein (EHD) family comprises four highly homologous human paralogs: EHD1, EHD2, EHD3, and EHD4 (see Figure 1C). These ATPases localize to distinct subcellular regions and regulate key steps of receptor trafficking [59, 60, 62] (see Figure 2). Impaired ATP‐binding EHD mutants exhibit delayed receptor recycling, highlighting the role of ATP binding and hydrolysis for functional recycling endosomes [6, 7, 8, 47, 63]. Among the four paralogs, EHD1 is the most extensively studied, likely due to its strong evolutionary conservation in invertebrates [6]. In mammals, EHD1 also mediates recycling of various types of internalized PM receptors such as transferrin [7], Major Histocompatibility Complex Class I (MHC‐I) [8], and β1 integrins [11, 31] and others, transporting them from the endosomal recycling compartment (ERC) to the PM.

By coordinating receptor transport, EHD proteins influence diverse physiological processes including cholesterol homeostasis [33], muscle development [39], potassium channel regulation [17], angiogenesis [56], and trafficking of the cystic fibrosis transmembrane conductance regulator (CFTR) [15]. Consistent with these roles, altered EHD expression or function has been associated with a range of human diseases, including metabolic and cardiovascular disorders [16, 24, 64], ciliopathies [5], and multiple cancers [25, 44, 65, 66, 67, 68]. However, the nature of this evidence varies considerably across disease contexts, ranging from direct human genetic findings to correlative tissue‐expression studies and mechanistic insights from cell and animal models.

### 2.1 Metabolic Regulation and Membrane Homeostasis

EHD proteins contribute to several aspects of metabolic regulation. EHD1 overexpression attenuates the downstream signaling by insulin‐like growth factor 1 receptor (IGF‐R1), potentially by affecting receptor internalization through interactions with SNAP29 and the adaptor protein 2 (AP‐2) complex [69]. In adipocytes, both EHD1 and EHD2 are required for insulin‐stimulated recycling of the GLUT4 glucose transporter [36, 70], linking EHD‐dependent trafficking to glucose uptake and insulin responsiveness [71, 72].

EHD proteins also participate in lipid homeostasis. EHD1 is involved in cholesterol regulation, potentially through direct or indirect effects on low‐density lipoprotein (LDL) receptor internalization [33]. EHD2 plays an important role in regulating adipocyte function and fat storage [73, 74, 75, 76]. At the PM, EHD2 stabilizes caveolae [77, 78, 79], and *Ehd2* knockout adipocytes show reduced fatty acid uptake [80]. EHD2 also associates with lipid droplets [73, 81, 82, 83, 84] promoting their degradation, with EHD2 depletion resulting in increased lipid droplet area [80, 83]. Together, these observations support a broader role for EHD proteins in coordinating membrane trafficking pathways related to glucose and lipid metabolism.

### 2.2 Immune Cell Trafficking and Host–Pathogen Interactions

Although EHD proteins have not been studied as extensively in immune cells, there is evidence that they function in both adaptive and innate immunity. By facilitating recycling of MHC‐I [8] and MHC‐II [13] receptors, EHD1 contributes to antigen presentation. Additionally, EHD1, EHD3, and EHD4 have been implicated in T‐cell responses and activation in CD4^+^ T‐cells, with the knockout of *Ehd1*, *Ehd3*, or *Ehd4* resulting in impaired T‐cell receptor (TCR) recycling of the TCR‐CD3 complex [58].

In innate immune cells such as developing macrophages, EHD1 is required for endocytic trafficking of Colony‐Stimulating Factor‐1 (CSF‐1) receptor [27]. *Ehd1* knockout bone marrow–derived macrophages (BMDMs) show reduced CSF‐1 receptor levels, defective downstream signaling, and impaired macrophage proliferation and migration [27].

EHD proteins also affect host–pathogen interactions. EHD4 depletion reduces Human Immunodeficiency Virus (HIV) infectivity [85], and in HIV‐infected macrophages, EHD3 is needed for macropinocytosis and its loss impairs phagophore‐lysosome fusion [86]. *Listeria monocytogenes* enters host intestinal cells at EHD2‐enriched PM sites [87], consistent with a role for EHD2 in bacterial uptake and spread. These findings show that EHD proteins regulate immune cell trafficking and are involved in the process of pathogen uptake during infection.

### 2.3 Neuronal and Synaptic Functions

EHD proteins regulate multiple aspects of neuronal trafficking and signaling. EHD1 facilitates recycling of AMPA‐type glutamate receptors from endosomes to the PM in post‐synaptic neurons, thereby strengthening synaptic transmission [14]. EHD1 also regulates neuronal excitability by recycling G protein‐activated inwardly rectifying K(+) (GIRK) channels [18]. EHD1 and EHD4 hetero‐oligomerize and regulate neuro‐glia cell adhesion molecule (NgCAM) internalization and axonal targeting, consistent with a role in transcytosis [29].

EHD proteins also support neurite growth and axonal transport. Nerve Growth Factor (NGF) stimulation recruits active Rab35 together with MICAL‐L1 and EHD1 to sorting endosomes [88], promoting transport of endosomes to growing axonal ends, thus facilitating neurite growth [88, 89]. EHD1 levels increase upon spinal cord injury [90], facilitating receptor transport for neurite growth and recovery [88, 89, 90], and EHD1 distribution on endosomes is maintained via interaction with the E3 ubiquitin ligase, Triad1, facilitating neurite growth during spinal cord injury [91]. In rat hippocampal neurons, EHD1 also negatively regulates exocytosis by competitively binding SNAPIN, preventing the latter from interacting with the SNARE protein SNAP‐25 [92].

Additional evidence implicates other EHD family members in neuronal function. EHD2 is upregulated in hemorrhagic rats [93], suggesting a mechano‐protective role in maintaining astrocyte PM turnover [94]. In the lamprey eel, the EHD1 and EHD3 ortholog (l‐EHD) is required for endocytic vesicle fission at neuronal synapses [95]. EHD4 levels also rise in rat neurons after NGF stimulation [54], and EHD4‐mediated macropinocytosis [96] is required for receptor transport, either supporting [96, 97] or inhibiting axonal growth [53]. At the neuromuscular junction (NMJ), both EHD1 and EHD4 localize to synaptic clefts, whereas the *Drosophila* ortholog Past1 is required for proper assembly of the subsynaptic membrane reticulum and for normal synaptic transmission [98]. Overall, these studies support broad roles for EHD proteins in synaptic trafficking, neuronal plasticity, and axonal remodeling.

### 2.4 Muscle Development and Repair

EHD1 and EHD2 play important roles in skeletal muscle development. Both proteins are required for myoblast fusion, a process essential for the formation of multinucleated muscle fibers [30, 39]. EHD2, which is highly expressed in developing muscle, interacts with myoferlin, a PM protein required for myoblast fusion, and mediates its recycling to the PM [39]. Accordingly, *Ehd2* knockout myoblasts accumulate intracellular myoferlin and exhibit markedly impaired fusion [39]. Both EHD1 and EHD2 also interact with Fer‐1‐like‐5 (Fer1L5), another myoferlin‐family member required for myoblast fusion; knockdown of either protein significantly reduces fusion [30].

In mature muscle, EHD proteins contribute to membrane organization and repair. Heterozygous *Ehd1* knockout mouse skeletal muscles show reduced muscle mass and poor contraction [99], partly due to faulty BIN1 accumulation on abnormal transverse‐tubule (T‐tubule) extensions [99, 100]. In skeletal muscle repair, EHD1 and EHD2, along with myoferlin and actin‐related proteins, localize to sites of tissue injury to promote membrane fusion and resealing of the damaged muscle fibers [40]. In primary human myotubes, however, EHD2 alone is required [101].

EHD proteins also regulate excitability in cardiac muscle. EHD1 recycles hyperpolarization‐activated cyclic nucleotide–gated (HCN) channels (HCN1–4), and its dysfunction contributes to cardiac arrhythmias [16]. EHD1 also interacts with phosphorylated Cx43, a gap junction protein, promoting its internalization, recycling, and retrograde transport from the Golgi to the Endoplasmic Reticulum (ER), and is essential for cardiac remodeling under ischemia [24]. EHD2 stabilizes K_ATP_ channels at the sarcolemma [41], whereas EHD3 maintains surface levels of T‐type Ca^2+^ channel (TTCC) and additional cardiac transporters [49] through interactions with ankyrin‐β [52, 102]. EHD4 may participate in the trafficking of the voltage‐gated Na^+^ transport channel in ventricular cardiomyocytes [57]. These findings highlight important roles for EHD proteins in both skeletal and cardiac muscle physiology.

### 2.5 Vascular and Endothelial Functions

EHD proteins also regulate endothelial trafficking and vascular function. EHD4 was first identified as a part of the extracellular matrix in human placenta [103]. It is highly expressed in human umbilical vein endothelial cells (HUVECs), where it facilitates endothelial cell migration via vascular endothelial (VE)–cadherin recycling, promoting angiogenesis [56], and is enriched in pancreatic islet vasculature [104]. Gene expression of EHD4 and EHD3 is also correlated with liver fibrosis, suggesting their potential role in angiogenesis [105, 106]. Additionally, EHD4 is required for the efficient recruitment of EHD1 to sorting endosomes [107], and EHD1 mediates recycling of the calcium‐activated potassium channel KCa2.3, crucial for endothelial polarity and blood pressure regulation [17].

EHD2 likewise contributes to vascular biology. In HUVECs, it promotes the internalization and transcytosis of delta‐like ligand 4, essential for Notch‐Delta signaling and angiogenesis [38]. At the PM, EHD2 stabilizes the eNOS receptor; its deletion causes patchy eNOS distribution and impaired blood vessel relaxation [42]. Taken together, these studies support important functions for EHD proteins in endothelial trafficking, angiogenesis, and vascular homeostasis.

### 2.6 Primary Ciliogenesis

**FIGURE 3:**
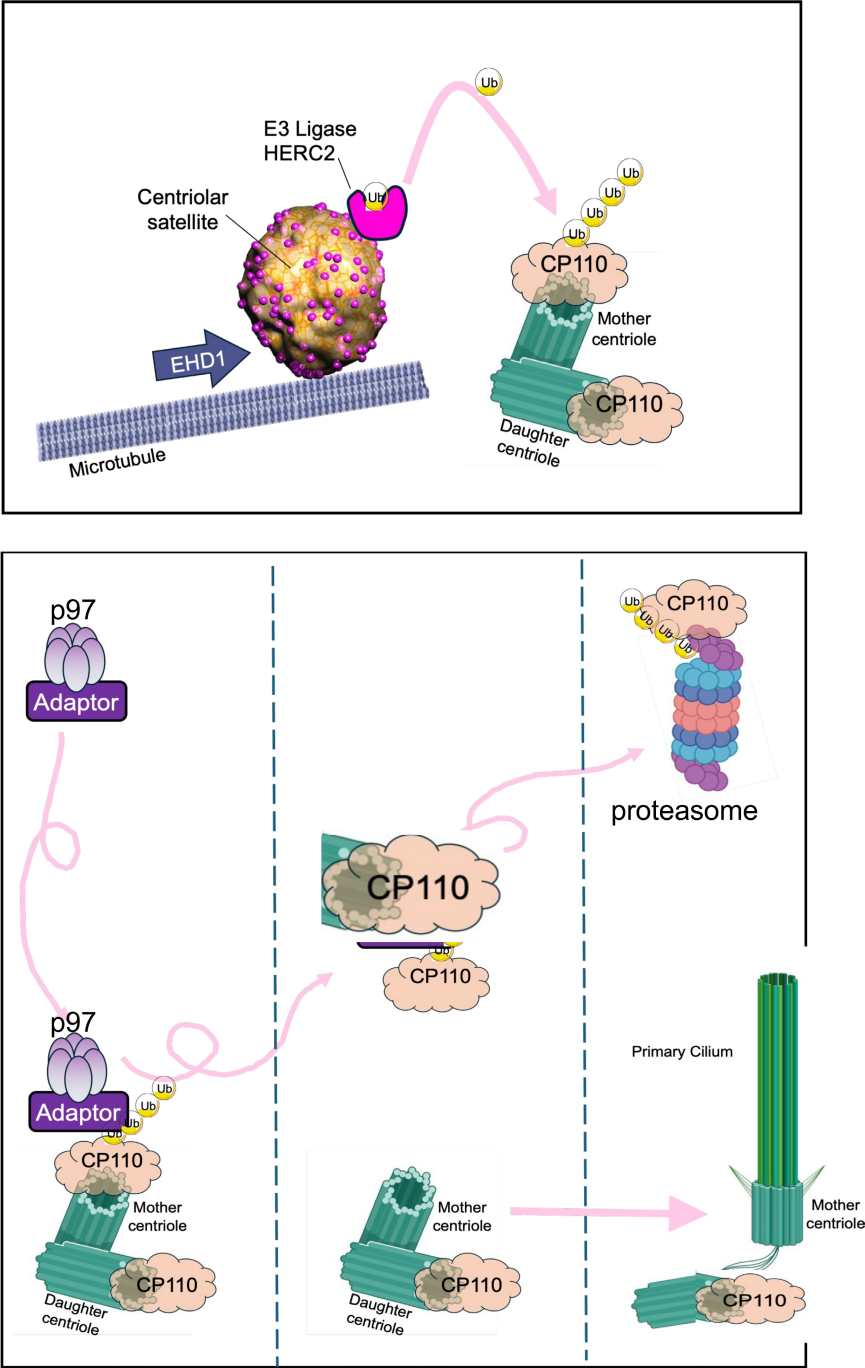
Proposed role for EHD1 in primary ciliogenesis. EHD1 promotes centriolar satellite movement from the periphery to the centrosome, potentially through modulation of microtubule growth. The centriolar satellites deliver the E3 ubiquitin ligase HERC2 to the mother centriole, where it ubiquitinates CP110. Upon CP110 polyubiquitination, the AAA ATPase valosin‐containing protein/p97 (p97) extracts CP110 from the mother centriole and targets it for proteasomal degradation, allowing the formation of the ciliary vesicle and growth of the axoneme, and ultimately generation of the primary cilium.

Three of the four EHD proteins play key roles in primary ciliogenesis (see Figure 3). The primary cilium is a key signaling organelle that controls cellular maturation and development, and failure to generate a primary cilium is the cause of severe developmental disorders known as ciliopathies [108]. Both EHD1 and EHD3 localize to the ciliary base, and depletion of either protein disrupts ciliogenesis in retinal pigment epithelial (RPE) and medullary epithelial cells [3]. EHD4 oligomerizes with EHD1 [55], and is also required for ciliogenesis in mouse fibroblasts [107].

These findings extend the role of EHD proteins beyond classical endocytic recycling and place them within the broader machinery that coordinates membrane remodeling at the centrosome and ciliary base. In the cochlea, EHD4 interacts with stereociliary membrane proteins involved in mechano‐transduction, although *Ehd4* knockout mice compensate through increased EHD1 expression and do not develop overt hearing defects [109, 110].

## 3 Disease Associations of EHD Proteins

Altered EHD expression, posttranslational modification, or function is associated with a range of human diseases, including metabolic and cardiovascular disorders, ciliopathies, inflammatory conditions, and cancer [5, 111, 112, 113, 114, 115]. However, the strength of this evidence varies substantially across disease categories. In most cases, the link to disease is based on altered expression, methylation, prognostic association, or mechanistic studies in cultured cells and animal models. Direct human genetic evidence remains limited, with the strongest example being the recently identified EHD1 founder mutation associated with a ciliopathy‐like syndrome [5]. Accordingly, the sections below distinguish, where possible, among direct human genetic findings, patient‐derived expression or association data, and mechanistic inferences from model systems.

### 3.1 Cardiometabolic Disease

Evidence linking EHD proteins to cardiometabolic disease is driven mainly by patient expression studies together with research on adipocytes and other model systems. In Type II diabetic patients, the EHD2 gene is methylated and suppressed [71, 116]. EHD2 protein levels in hypoxic adipocytes contribute to insulin resistance [72]. EHD2 is also suppressed in obesity [116], and both EHD2 and caveolin‐1 levels are reduced in insulin‐resistant adipocytes [72]. These observations implicate altered EHD2 expression in metabolic dysfunction, although direct causal human genetic evidence is lacking.

Mechanistically, both EHD1 and EHD2 are required for insulin‐stimulated recycling of GLUT4 [36, 70], providing a plausible link between EHD dysfunction and impaired glucose uptake. EHD2 additionally regulates caveolar stability, fatty acid uptake, and lipid droplet turnover [73, 74, 75, 76, 77, 78, 79, 80, 81, 82, 83, 84], while EHD1 contributes to cholesterol homeostasis, potentially through its impact on LDL receptor trafficking [33]. Thus, although the majority of evidence is correlative, this supports a functional role of EHD‐dependent membrane trafficking in glucose and lipid homeostasis.

### 3.2 Cardiovascular Disease

Evidence for EHD involvement in cardiovascular disease similarly combines human association data with strong mechanistic support from cellular and animal models. EHD3 levels are elevated in heart failure [64], and EHD2 synthesis is dysregulated during hematopoiesis in patients with excessive platelet formation [117]. Elevated EHD2 levels have also been reported in hypertension drug‐treated rats [118]. These observations support cardiovascular relevance but lack direct causation in humans.

Mechanistic studies provide a stronger framework for interpretation. EHD1 regulates recycling of HCN channels and trafficking of phosphorylated Cx43 [16, 24], implicating it in arrhythmia and ischemic remodeling. EHD2 stabilizes KATP channels at the sarcolemma, and ATP‐deficient EHD2 mutants increase channel internalization and reduce conductance in ventricular myocytes [41]. EHD3 controls trafficking of T‐type Ca2+ channels, β1‐adrenergic receptors, Na/Ca exchangers, and l‐type calcium channels, and cardiac depletion causes abnormal conductance, bradycardia, and defective ventricular function [49, 52]. EHD1, EHD2, and EHD4 also participate in endothelial trafficking pathways relevant to vessel relaxation and angiogenesis [38, 42, 56]. Overall, these findings support a role for EHD proteins in cardiovascular physiology and disease, despite the lack of direct genetic evidence.

### 3.3 Immune, Inflammatory, and Infectious Disease Associations

Links between EHD proteins and immune or infectious disease are currently supported mainly by altered expression in disease settings and by mechanistic studies in immune cells. For example, macrophages associated with coronary heart disease show elevated EHD1, which is required for TNFR2 recycling and maintenance of IL1‐β and TNF production [28]. EHD2 expression increases in SARS‐CoV‐2‐infected lung cells [119], and EHD2 [120] and EHD4 [121] are elevated in obstructive pulmonary inflammation. EHD4 secretome levels are also increased in pediatric pneumonia [122]. These findings suggest that EHD proteins may be engaged in inflammatory remodeling, but they do not establish causation.

Functional studies nonetheless provide mechanistic context. EHD1 regulates antigen presentation through MHC‐I and MHC‐II recycling [8, 13] and is required for macrophage CSF‐1R trafficking [27]. EHD1, EHD3, and EHD4 also contribute to TCR recycling [58]. In viral settings, EHD4 depletion reduces HIV infectivity [85], EHD3 supports macropinocytosis and phagophore‐lysosome fusion in HIV‐infected macrophages [86], and EHD2‐enriched PM domains facilitate *Listeria monocytogenes* entry into cells [87]. Accordingly, evidence places EHD proteins at the intersection of immune trafficking and host–pathogen interactions.

### 3.4 Neurologic and Neuropsychiatric Associations

The neurologic disease literature is more heterogeneous and remains largely correlative to date. Elevated EHD3 levels have been connected to cognitive function in autism patients [123], genetic alterations in EHD3 have been reported in females with major depressive disorder [124, 125, 126], and EHD4 variation has been associated with Progressive Supranuclear Palsy, a primary taupathy [127]. EHD4 is also upregulated in aged post‐stroke rat brains [128], and elevated EHD4 protein levels have been reported in alcohol‐drinking males [129]. These studies suggest potential neurologic relevance.

Studies done in neurons provide biological context for these associations. EHD1 regulates recycling of AMPA receptors and GIRK channels [14, 18], EHD1 and EHD4 control NgCAM trafficking [29], and EHD1 is required for neurite growth after NGF stimulation and spinal cord injury [88, 89, 90, 91]. Additional model‐organism studies, including work on Past1 at the *Drosophila* NMJ [98] and l‐EHD at lamprey synapses [95], further support conserved roles in neuronal membrane trafficking. Overall, these findings are consistent with roles for EHD proteins as regulators of neuronal function, but the direct connection to specific human neurologic diseases remains provisional.

## 4 Cancer

Among non‐ciliopathy disease categories, the most extensive literature linking EHD proteins to pathology can be found in various cancers. However, this evidence is dominated by altered expression, prognostic association, and mechanistic studies in model systems rather than by human genetic causation. As a result, EHD proteins are best viewed at present as modulators of tumor‐associated membrane trafficking pathways whose effects are often context dependent.

### 4.1 EHD1 in Cancer

EHD1 is the best studied family member in cancer, consistent with the broader principle that cancer cells frequently reprogram membrane‐trafficking pathways [130], including those regulated by EHD proteins [60]. High levels of EHD1 expression correlate with reduced disease‐free survival, reduced overall survival, and poor response to chemotherapy in multiple tumor types [22, 25, 26, 65, 131, 132, 133, 134]. Mutations in EHD1 have also been linked to malignant papillary mesothelioma [135] and monoclonal plasma cells in POEMS syndrome [136], although these findings do not yet establish a broadly causal oncogenic role.

Mechanistically, EHD1 promotes trafficking of multiple receptors and signaling proteins with known roles in tumor progression. It mediates transport of IGF‐1R and EGFR from the Golgi to the PM, possibly through hybrid secretory‐endosomal routes [22, 23, 65, 66]. It also promotes recycling of CD44, β1‐integrin, and β2‐adrenergic receptors, thereby supporting stemness, motility, VEGF signaling, and angiogenesis [11, 25, 26, 32]. Elevated CD44 surface levels suppress Hippo signaling, promoting YAP nuclear translocation, stemness, and tumor growth [25], whereas EHD1 depletion reduces CD44 and stemness markers [26], impairing migration and invasion [25, 137]. EHD1 also promotes β1‐integrin recycling [11] and regulates Src transport to the PM [31], which is required for focal adhesion turnover and cell motility; accordingly, EHD1 depletion causes β1‐integrin accumulation on endosomes and Src aggregation in the ERC, resulting in impaired migration [11, 31]. In addition, EHD1 recycles β2‐adrenergic receptors, supporting VEGF signaling and angiogenesis in growing tumors [32]. EHD1 depletion attenuates Akt/mTOR signaling, lowers HIF2α and matrix metalloprotease output, reduces β‐catenin activation and glucose uptake, and decreases tumor proliferation and invasiveness [67, 138, 139, 140]. High EHD1 expression also correlates with TGFβ‐1 receptor expression, consistent with a role in TGFβ receptor recycling that could enhance tumor proliferation and survival [141]. The interaction of EHD1 and EHD4 with Phostensin at endosomes regulates transferrin receptor recycling [140, 142], providing an additional trafficking axis that may influence tumor progression. Moreover, EHD1 loss prevents 14‐3‐3ζ dimerization and enhances its phosphorylation, attenuating β‐catenin activation and reducing glucose uptake and tumor growth [67], while EHD1 depletion also reduces CDK2 phosphorylation, thereby suppressing proliferation [139]. EHD1 also contributes to chemoresistance and immune evasion. EHD1 and EHD2 have both been implicated in chemoresistance [44, 143], and EHD1 depletion increases intracellular cisplatin and restores drug sensitivity [134]. Conversely, EHD1 upregulation promotes PI3K/Akt signaling and gefitinib resistance, whereas its suppression restores gefitinib sensitivity [144, 145]. EHD1 also recycles Programmed Death‐Ligand 1 (PDL1), enabling immune evasion, and its depletion improves anti‐PD‐L1 immunotherapy efficacy [146]. Furthermore, interaction of N6‐methyladenosine‐modified EHD1 mRNA with YTH N6‐methyladenosine RNA‐binding protein 1 stabilizes EHD1 expression and promotes tumor progression [146]. Overall, the evidence strongly favors a pro‐tumorigenic role for EHD1 in most contexts.

### 4.2 EHD2 in Cancer

The role of EHD2 in cancer is more context dependent. High EHD2 expression correlates with poor survival in some cancers, including clear cell renal cell carcinoma and glioma, and may serve as a biomarker [44, 147, 148, 149, 150, 151, 152]. In contrast, tumor‐suppressive roles have been described in lung adenocarcinoma and hepatocellular carcinoma [45, 153, 154].

Functional studies reflect this duality. In some cases, EHD2 depletion impairs migration and invasion and reduces CD44 levels and stemness markers, suggesting a pro‐tumorigenic role [147, 148]. EHD2 also regulates ORAI1 surface abundance and calcium homeostasis [43], and its locus can generate a circular RNA that promotes glucose uptake and metabolic activity in lung adenocarcinoma [155]. EHD2 is also a HIF‐2α target that may serve as a biomarker for clear cell renal cell carcinoma [149], and knockout of HIF‐1α or EHD2 represses hypoxia‐induced macropinocytosis, thereby impeding hypoxic hepatocarcinoma cell growth [156]. Suppression of EHD2 by lncRNA TUSC8 inhibits migration [157], although gain‐of‐function mutations have also been reported to promote tumor spread [158]. In other settings, however, loss of EHD2 enhances migration, invasion, metastasis, and epithelial‐mesenchymal transition, whereas EHD2 upregulation inhibits tumor progression [153, 159]. Additional studies support tumor‐suppressive functions for EHD2: in resting cells EHD2 is bound to myoferlin, whereas IL‐6 stimulation promotes dissociation, freeing myoferlin to bind activated STAT3 and chaperone it to the nucleus, consistent with a negative regulatory role for EHD2 [160]. Ese3 transcription factor‐mediated EHD2 downregulation increases proliferation and tumor volume [161], EHD2 overexpression reduces migration, invasion, and epithelial‐mesenchymal transition while promoting cell‐cycle arrest and apoptosis in colon cancer [162], and EHD2 is negatively regulated at the transcriptional level by PIGX in proliferating breast cancer [163]. The positive relationship between EHD2 and E‐cadherin is notable in that it supports a tumor‐suppressive interpretation in several epithelial cancers [44, 45, 46, 164]; indeed, EHD2 depletion reduces E‐cadherin levels and promotes cancer cell migration and epithelial‐mesenchymal transition [44, 45, 46]. Thus, EHD2 may act either as an oncogenic or tumor‐suppressive factor, depending on cellular context.

### 4.3 EHD3 and EHD4 in Cancer

EHD3 is abundantly expressed in small‐cell lung cancer but is markedly downregulated or epigenetically silenced in multiple other cancers [111, 112, 113, 114, 115]. EHD3 also has important effects on receptor signaling. For example, EHD3 upregulation can stabilize EGFR by reducing its ubiquitination, without affecting Akt/ERK activation [50]. In addition, EHD3 mediates β3‐integrin recycling, and its depletion impairs receptor recovery and adhesion [51]. In gastric cancer models, EHD3 depletion reduces proliferation, migration, invasion, epithelial‐mesenchymal transition, and tumor size [68]. However, in glioma, EHD3 overexpression suppresses proliferation, migration, and tumor growth [114]. Thus, as with other EHD family members, the effects of EHD3 in cancer appear strongly context dependent.

EHD4 also appears to have tumor‐type‐specific roles. It has been reported to be protective in colorectal cancer [165], but elevated in hepatocellular carcinoma [166], and it may also stabilize an anti‐apoptotic microRNA required for tumor progression. Overall, a complex pattern has emerged for EHD proteins in cancer, with family members exhibiting context‐dependent oncogenic or tumor‐suppressive functions.

### 4.4 Ciliopathies

Ciliopathies provide the strongest direct human genetic evidence linking EHD dysfunction to disease. The clearest example is a founder mutation in EHD1, R398W, which causes high‐frequency hearing loss, proteinuria, and polycystic kidney disease, a phenotype consistent with a ciliopathy [5]. In mice, the same pathway is linked to defects in spermatogenesis and male infertility [110], further supporting the physiological significance of EHD1‐dependent ciliary function.

This human genetic evidence is reinforced by mechanistic studies. EHD1 and EHD3 localize to the base of primary cilia, and depletion of either protein disrupts ciliogenesis in retinal pigment epithelial and medullary epithelial cells [3, 4, 167, 168]. EHD4 oligomerizes with EHD1 [55] and is also required for ciliogenesis in mouse fibroblasts [107]. Although altered EHD3 levels have been associated with diabetic retinopathy in a patient subpopulation [169], and EHD4 participates in cochlear membrane trafficking [109], these links remain less definitive than the EHD1 R398W syndrome [5]. Overall, the ciliopathy field currently provides the most compelling example of a direct disease connection for the EHD family.

## 5 EHDs in Animal Models

### 5.1 Mus musculus

*Mus musculus* encodes four EHD homologs, EHD1, EHD2, EHD3, and EHD4, that share over 70% amino acid sequence identity [1] (see Figure 1C). Mouse EHD1 displays 99% amino acid sequence identity with its human ortholog, making the mouse an ideal model for studying EHD1's physiological functions.

EHD1 is ubiquitously expressed in mouse tissues, with particularly high levels in developing male germ cells of the testis, consistent with its role in spermatogenesis [1, 170]. Mutant *Ehd1* (R398W) knock‐in mice exhibit defective acrosome formation [110]. Consistent with a role for EHD proteins in the testis, *Ehd4* knockout mice display abnormal spermatogenesis [171]. EHD1 levels are doubled in these knockout mice, indicating partial compensation for EHD function during male gonadal development [171]. EHD3 in Leydig cells is also required for testosterone secretion in mice testes [172]. EHD3 is highly expressed in kidney glomerular endothelial cells [173, 174], where it is essential for maintaining effective filtration. In diabetic (obese) mouse models, reduced EHD3 expression correlates with increased glomerular fenestrations and impaired filtration [175]. *Ehd3* knockout mice have compensatory upregulation of EHD4 [176], while double *Ehd3* and *Ehd4* knockout mice develop severe proteinuria [176].

Mouse models have also been used to elicit the role of EHD2 in adipocytes for fat storage [80, 116]. EHD2 regulates lipid droplet size and is essential for insulin‐mediated signaling and glucose uptake [72, 177, 178, 179]. EHD2 is also required for the stability of eNOS receptors at the PM, with *Ehd2* knockout mice displaying impaired blood vessel relaxation [42]. However, EHD2 is dispensable for eNOS‐dependent calcium release in vascular smooth muscle cells of aged mice [180]. Additionally, EHD1 and EHD3 regulate the dynamic transport of β‐secretase in hippocampal neurons, which is dysregulated during Alzheimer's disease progression [181].

During embryogenesis, EHD1 is expressed in the developing heart, occipital lobes, and limb buds [1], suggesting roles in early organogenesis. While some *Ehd1* knockout studies report no overt phenotype in adults [9], others observed partial embryonic lethality [170]. These discrepancies likely reflect genetic background differences or compensation by other EHD proteins. Notably, transferrin receptor recycling is significantly delayed in *Ehd1* knockout mice, mirroring the receptor recycling defects seen in human cells [9, 10]. Mutant *Ehd1* knock‐in mice, with the same R398W founder mutation, exhibit impaired uptake of low molecular weight dextran in proximal convoluted tubules, leading to proteinuria [5]. This mislocalized and inactive EHD1 mutant causes hearing loss in the inner ear, underscoring EHD1's critical roles in endocytic trafficking and sensory function [5].

### 5.2 Danio rerio

**FIGURE 4:**
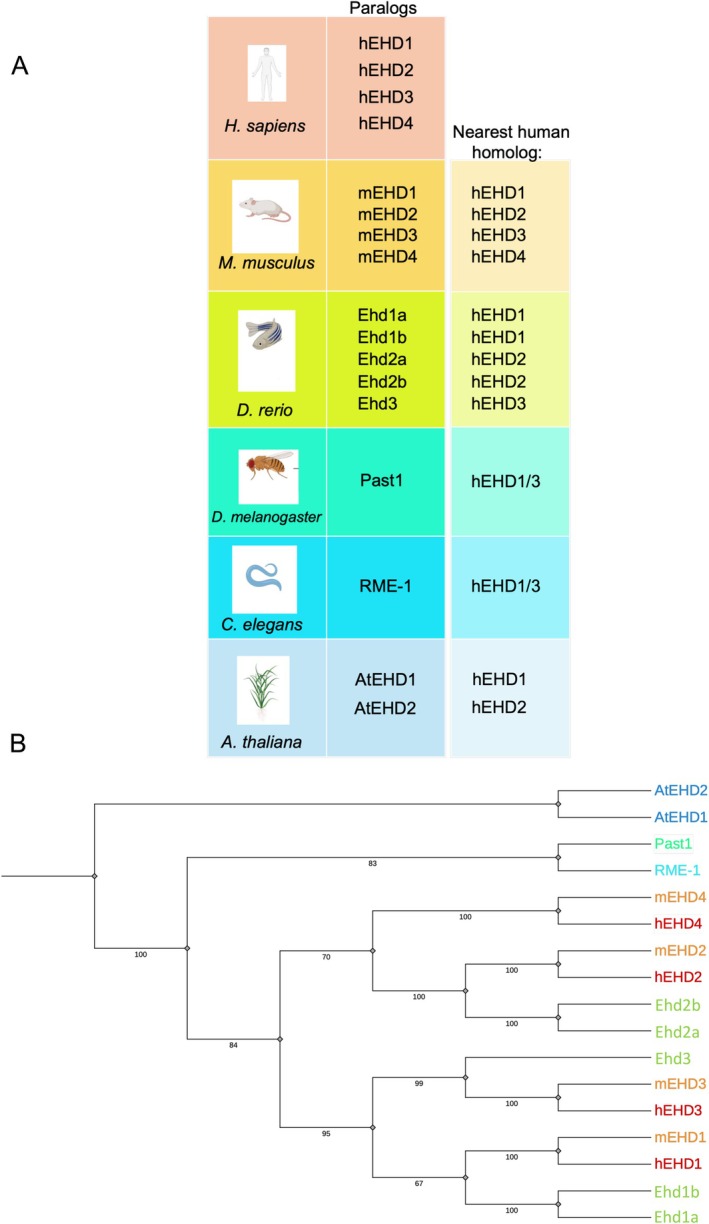
C‐terminal EHD paralogs in humans and other species. (A) EHD proteins classified by species and related to their nearest human homolog. (B) A dendrogram was generated to show similarity of EHD homologs. Protein sequences were collected from NCBI and aligned using MUSCLE, the alignment results were entered into IQ‐Tree, and iTOL was used to visualize the results. This process was streamlined using the Phylogenetic_Analysis_Protein_Sequences pipeline created in Colab (https://github.com/Ash100/Alignment). Bootstrap values indicating branch confidence are shown.

*Danio rerio* expresses five EHD homologs: ehd1a, ehd1b, ehd2a, ehd2b, and ehd3, reflecting gene duplication events. Ehd1a/b paralogs are highly similar to each other (89% identity) and closely related to the human EHD1 ortholog, while ehd3 most closely resembles the human EHD3 ortholog (see Figure 4A,B). Knockdown of ehd1a/b causes synergistic embryonic lethality, exacerbated by concurrent ehd3 loss, indicating distinct but complementary roles in organism survival [3].

Functional analyses show that ehd1 and ehd3 play cooperative but tissue‐specific roles in ciliogenesis and endocytic trafficking [3]. Both ehd1 and ehd3 are required for photoreceptor cilium formation, whereas ehd1 alone is critical for kinocilium formation in otic cells, consistent with its higher expression in the ear [3]. At neuromasts, ehd1 is essential, whereas ehd3 makes a partial contribution to cilia formation, and defects are rescued by human EHD1 or EHD3, but not by EHD4, demonstrating strong functional conservation of EHD proteins from mammals to zebrafish [3]. Additionally, ehd1b is highly expressed in kidneys, and *ehd1a/b* knockout embryos show reduced endocytic uptake in the pronephric tubules, demonstrating conserved renal trafficking functions [5]. Consistent with human cells and mouse models, loss of ehd2a/b in zebrafish embryos promotes dysmorphic sprouting in blood vessels due to impaired delta‐notch signaling [38].

### 5.3 Drosophila melanogaster

*Drosophila melanogaster* encodes a single EHD ortholog Past1 (*p*utative *a*chaete *s*cute *t*arget 1), originally identified as one of the targets for the *achaete‐scute* gene complex. Past1 was initially cited as phylogenetically closest to the mammalian EHD2 ortholog [182], but displays greater amino acid identity with both EHD1 and EHD3 (see Figure 4A,B). It has two transcripts: the longer is expressed throughout development in both sexes, while the shorter is male‐specific, expressed in the third larval stage and enriched in testes, indicating germ‐line specific regulation [182].

Past1 deletion mutants show defective sperm individualization [182], consistent with mouse models [110, 171, 176], as well as abnormal ovary morphology, and ~20% slower development, partially due to interactions with the Notch receptor [182]. Past1 deletion mutants also exhibit lower survival at high temperatures [182], similar to temperature‐sensitive *shibire* mutants [183].

Past1 may regulate developmental processes, including eye development, potentially through effects on Numb‐associated endocytic trafficking [184], with Past1 deletion mutants showing defective photoreceptors and cone cell differentiation [185]. At the NMJ, Past1 interacts with Syndapin to stabilize tubulovesicular structures, and NMJ in *Past1* knockout flies display collapsed synaptic boutons [98]. In cultured cells, Past1 localizes mainly to the PM and mediates endocytosis in garland cells and larval nephrocytes [182]. Additionally, Past1 regulates endosome‐to‐Golgi retrograde trafficking required for salivary granule maturation [186], highlighting its diverse roles in membrane trafficking during development.

### 5.4 Caenorhabditis elegans

*Caenorhabditis elegans* encodes a single EHD ortholog, RME‐1 (*r*eceptor‐*m*ediated *e*ndocytosis‐1), sharing 67% identity with human EHD1 and similar identity with human EHD3 [6] (see Figure 4). RME‐1 was first identified in a genetic screen where mutant oocytes showed abnormal uptake of fluorescently labeled yolk proteins [187]. A recent study also indicates that RME‐1 is required for efficient secretion of vitellogenins, the lipoprotein precursors of yolk proteins, to the pseudocoelom [188]. The *Rme‐1* gene in *C. elegans* has five splice isoforms: the shortest is expressed at all developmental stages, while the longest appears weakly in the second larval stage and increases with age, peaking in adults [6]. *Rme‐1* deletion mutants or knockdown worms show no significant embryonic or larval lethality, although brood sizes of deletion mutants are smaller than wild‐type counterparts [6].

RME‐1 studies revealed an evolutionarily conserved structural and functional role for EHD proteins in receptor trafficking [6]. Point mutants display defective endocytosis in coelomocytes, where the G81R mutation in the ATP‐binding loop hampers nucleotide binding [6, 63]. Deletion mutants accumulate intestinal vacuoles starting at the fourth larval stage, increasing in size and number with age, reflecting RME‐1's role in basolateral recycling of receptors [6]. Indeed, RME‐1 interacts with *C. elegans* ALX‐1 to control recycling of basolateral cargo in the worm intestine [189]. Additional studies further support a role for RME‐1 within the basolateral recycling pathway, showing that RAB‐10 functions upstream of RME‐1 in the intestine, whereas loss of the Numb homolog NUM‐1 can bypass the requirement for RME‐1, consistent with pathway‐level regulation of RME‐1‐dependent recycling [190, 191].

In worms, RME‐1 also interacts with AMPH‐1, a bar domain‐containing protein that may limit RME‐1 ring assembly on membrane tubules, potentially contributing to the regulation of endosome fission [192]. Moreover, RME‐1 is recruited to arrested endocytic intermediates induced upon loss of filamentous actin [193]. Overall, studies in *C. elegans* largely support a conserved role for RME‐1 in endocytic recycling that is consistent with the functions of its mammalian homologs.

## 6 Plants

Plant EHD orthologs have conserved roles in membrane trafficking and regulate distinct physiological processes. *Arabidopsis thaliana* encodes two EHD‐like proteins, AtEHD1 and AtEHD2, that share ~74% similarity with mammalian EHD1 and EHD2 orthologs, respectively, although it should be noted that the plant AtEHD proteins contain their EH domains at the N‐terminus of the protein, unlike all other EHD proteins [37]. Both proteins localize to the PM, suggesting roles in endocytic and vacuolar trafficking. AtEHD1 silencing accelerates flowering [37], likely via altered hormone or stress signaling, and impaired endocytic recycling. AtEHD2 overexpression inhibits internalization of receptors like transferrin and leucine‐rich repeat receptor‐like protein (LeEix2), revealing differential regulation of membrane trafficking by AtEHD proteins [37]. AtEHD2 plays a crucial role in pathogen defense by inhibiting LeEix2 internalization, following binding to the fungal ligand ethylene‐induced‐xylanase, thus preventing downstream ethylene biosynthesis [34]. The coiled‐coiled and nucleotide‐binding domain of AtEHD2 binds and prevents LeEix2 receptor internalization [194]; mutants lacking these domains exhibit altered actin organization at the PM, reduced ethylene production, and compromised pathogen defense [194]. Additionally, SUMOylation regulates AtEHD2 distribution and function in anti‐fungal defense [35]. Mutation of the SUMOylation site in AtEHD2 promotes LeEix2 endocytosis and thereby mitigates anti‐fungal defense response [35].

## 7 Physiological Functions of EHDs

### 7.1 Receptor Recycling

Upon internalization, PM receptors are delivered to the early or sorting endosome (EE) [195, 196]. The EE is a sorting station from which receptors and lipids are shunted to different trafficking routes, including late endosomes (LEs) and lysosomes for degradation, retrograde trafficking to the trans‐Golgi, or recycling back to the PM [61, 197]. These trafficking pathways are tightly regulated, and impaired receptor trafficking has been linked to Alzheimer's disease, cardiovascular disease, various cancer types, and other illnesses [198, 199]. Receptors destined for the PM may be recycled directly from the EE, a process termed fast recycling, or they can initially be trafficked to a perinuclear endocytic recycling compartment (ERC), in a process referred to as slow recycling [61]. All four EHD paralogs have been identified as regulators of various steps of these trafficking pathways [60] (Figure 2).

Studies with the *C. elegans* homolog RME‐1 provided the first evidence that the EHD proteins regulate endocytic trafficking [6, 7]. In Chinese Hamster Ovary cells, EHD1 mutants impaired transferrin (Tf) receptor recycling and caused accumulation of cargo in the ERC [7]. Subsequent studies with the human homolog showed that EHD1 regulates the recycling of MHC‐I receptors from tubular recycling endosomes (TREs) [8]. Although overexpression of a dominant‐negative EHD1 mutant did not affect the internalization of cargos, it disrupted recycling of both clathrin‐dependent (Tf) and clathrin‐independent (MHC‐I) receptors [7, 8]. Since these initial studies, many receptors have been identified as being regulated by EHD1, including the cystic fibrosis transmembrane conductance receptor [15], β1 integrin [11], GLUT4 transporters [36], CD59 [12], MHC class II molecules [13], AMPA‐type glutamate receptors [14], and others (Table 1).

**FIGURE 5:**
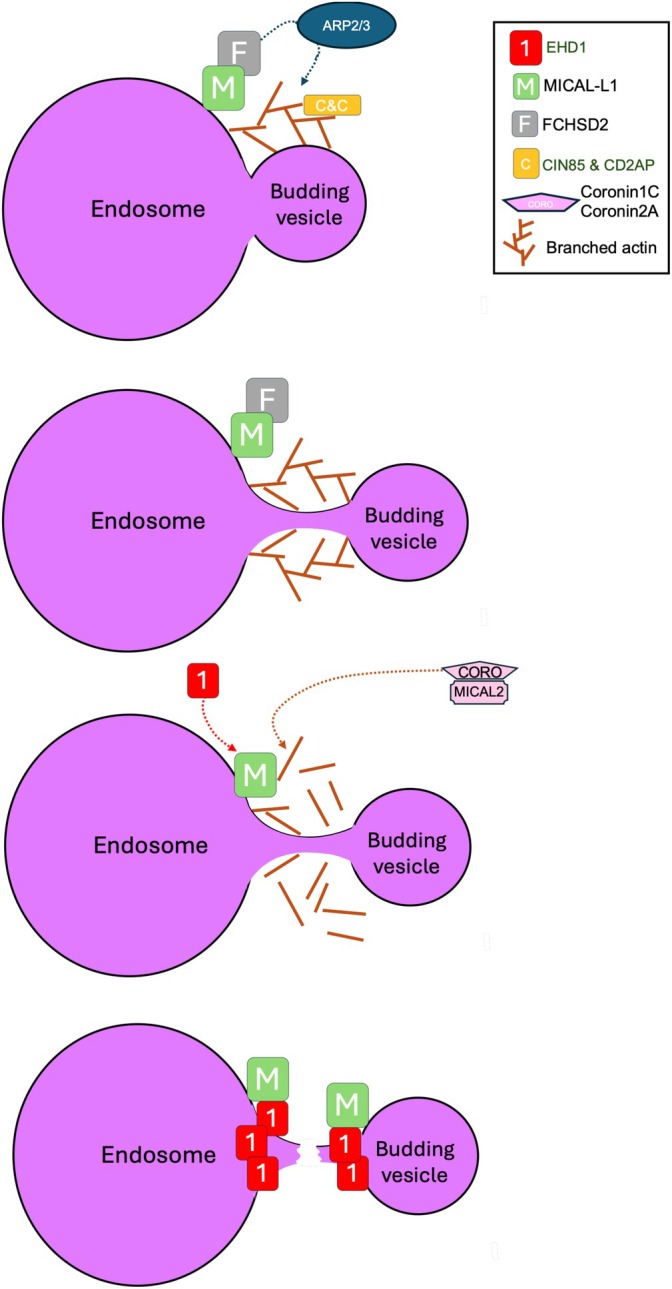
Proposed role for EHD1 in mediating membrane fission. After cargo sorting, endosome budding is facilitated by BAR domain proteins and actin remodeling. MICAL‐L1 binds to membranes and recruits FCHSD2, which subsequently activates ARP2/3 to generate branched actin. MICAL‐L1 also recruits CIN85 and CD2AP, which may support removal of actin capping protein (not shown) to further promote branched actin. Once the vesicle is generated, to undergo fission Coronin1C and Coronin2A are recruited to debranch actin and clear the membrane for fission. EHD1 is recruited by MICAL‐L1. EHD1 undergoes oligomerization and promotes fission of the budding vesicle through its ATP hydrolysis.

Beyond its role at the ERC, EHD1 has also been localized to RAB35‐positive EEs, where it regulates cargo trafficking from EEs to recycling endosomes in complex with CRMP2 and MICAL‐L1 [200, 201, 202]. Moreover, loss of EHD1 function leads to enlarged EEs [19, 203], elongation of TREs [204], accumulation of cargos in endosomes, and impaired receptor recycling [7, 8]. These findings raised the possibility that EHD1 plays a role in fission at endosomes (see Figure 5).

The four EHD paralogs localize to different intracellular compartments and regulate different points of the endocytic trafficking pathway [8, 205, 206] (Figure 2). EHD1 and EHD3 have similar subcellular distribution patterns [8, 205]. While both EHD1 and EHD3 localize to EEs and TREs, EHD1 is enriched at TREs, and EHD3 has increased EE localization [8, 47, 62, 200, 205]. EHD1 has also been implicated in retromer‐based trafficking from the EE to the Golgi [207, 208, 209]. In contrast with EHD1 at TREs, EHD3 stabilizes tubular recycling endosomes [210]. Unlike EHD1 depletion, EHD3 knockdown leads to cargo accumulation in EEs rather than the ERC, highlighting EHD3 as a regulator of cargo trafficking from EE to ERC [47, 206]. EHD4 localizes to EEs but can also be observed on TREs [55, 206]. EHD4 depletion leads to EE enlargement, consistent with impaired trafficking and/or endosomal maturation and fission, and functional assays support a role for EHD4 in recycling to the PM as well as transport of cargo from EEs to LEs [55].

Although EHD1, EHD3, and EHD4 perform both distinct and overlapping functions in endocytic trafficking, heterodimerization among these proteins adds a level of regulatory complexity [47, 203, 206]. Indeed, EHD4 depletion reduced EHD1 recruitment to EEs and TREs, suggesting a cooperative role in regulating cargo trafficking [55, 203]. EHD1 and EHD3 heterodimerization has been proposed to facilitate cargo transport from EEs to TREs [47]. EHD2, however, has not been implicated in receptor recycling, but is a regulator of caveolae‐mediated endocytosis.

### 7.2 Caveolae Stabilization

Caveolae are small invaginations in the PM that participate in myriad cellular processes. These include endocytosis [211, 212, 213, 214], fatty acid uptake [215], mechanoprotection [216, 217, 218, 219], lipid homeostasis [220, 221, 222, 223], regulation of membrane composition [224, 225], and cellular signaling [226, 227, 228]. Assembly of caveolae occurs in cholesterol, sphingomyelin, and ceramide‐dense microdomains of the PM and is driven by the oligomerization of caveolin and cavin proteins [229, 230, 231]. Caveolin proteins (caveolin‐1–3) comprise the caveolar membrane coat and promote caveolar bulb curvature [232, 233, 234, 235], whereas cavin proteins are recruited from the cytoplasm to generate caveolae and stabilize the invaginated bulbs [234, 236, 237, 238].

EHD2 was first discovered in caveolar fractions via ultracentrifugation and was later shown to colocalize at PM puncta with caveolin‐1 and cavin‐1 [84, 239, 240, 241]. Whereas caveolin‐1 is evenly distributed around the invaginated structure, EHD2 specifically localizes closer to the neck of the invagination at the PM [241, 242, 243]. PM association of EHD2 is regulated by phosphatidylinositol 4,5‐bisphosphate levels, which are enriched at caveolae [244, 245]. EHD2 binds and hydrolyzes ATP, which regulates its dissociation from caveolae. Expression of EHD2 mutants with impaired ATPase activity significantly reduced the number of caveolae at the membrane [77, 239]. However, EHD2 must oligomerize in order to regulate caveolae [77, 241].

EHD2 depletion does not affect the formation or phenotype of caveolae in cells, suggesting a role in caveolar dynamics rather than their generation [239, 240, 241]. Indeed, caveolae in EHD2‐depleted cells have increased caveolae internalization and motility, supporting a role for EHD2 in caveolar stabilization at the PM [78, 239, 241, 246, 247]. EHD2 likely regulates caveolae mobility in cooperation with the actin cytoskeleton [241], and the EHD2 interaction partner EHBP1 is an actin regulatory protein that localizes to the caveolar neck, thus providing a basis for caveolae‐actin tethering [38, 245, 248]. Another EHD2 interactor, Syndapin2/PACSIN2, also localizes to caveolae to cooperatively promote caveolae stability and to prevent scission [240, 247].

The mechanism for EHD2 stabilization of caveolae has not fully been elucidated. However, it has been hypothesized that EHD2 increases the diameter of the caveolar neck to prevent lipid phase separation or the assembly of proteins required for protein‐mediated scission [247, 249, 250, 251]. EHD2 has a slow nucleotide hydrolysis rate, which may contribute to slow caveolar dynamics by increasing EHD2‐caveolae retention time [239, 252]. Lastly, diffusion of glycosphingolipids and cholesterol into caveolae stimulates endocytosis. It has been proposed that EHD2 modulates caveolae stability by regulating the diffusion of these lipids into caveolae [233, 253, 254, 255, 256].

Caveolae are important mechanosensory structures on the PM that adapt different conformations according to changes in membrane tension [216, 236]. High membrane tension leads to the flattening of caveolae, which is reversed upon release of mechanical stress [216, 236]. EHD2 depletion reduced caveolae recovery from mechanical stress, indicating it is required for re‐stabilization of caveolae after stress [257], and EHD2 undergoes SUMOylation upon mechanical stress, releasing it from the PM and facilitating nuclear translocation. EHD2 is also involved in caveolae mechanotransducer function [257].

While EHD2 has been the most extensively studied EHD protein at caveolae, other EHDs might have compensatory functions. Upon EHD2 knockdown, EHD1 and EHD4 localized to caveolae [251]. Depletion of EHD1, 2, and 4 decreased caveolae clustering on the membrane, increased caveolae mobility, significantly reduced neck diameter, and increased cellular sensitivity to PM rupture [251]. Elucidating the roles of EHD1 and 4 on caveolae dynamics requires further investigation.

### 7.3 EHD Proteins in Primary Ciliogenesis

Over the past decade, EHD proteins (primarily EHD1) have been implicated in the regulation of primary ciliogenesis (see Figure 3). The primary cilium (PC) is a single, immotile, antenna‐like organelle that is generated under conditions of stress to equip the cell to navigate various environmental stressors [168]. Structurally, the PC is a microtubule axoneme that extends from the mother centriole (m‐centriole), also known as the basal body [258, 259]. The axoneme is surrounded by a ciliary membrane likely derived from endocytic vesicles. This membrane fuses with the lipid bilayer at the PM to form a distinct lipid environment that is contiguous with, yet compositionally distinct from, the PM. Protruding into the extracellular environment, the PC is enriched in ion channels, G protein–coupled receptors, and receptor tyrosine kinases, which contribute to higher‐order processes including signal transduction, mechano‐transduction, and nutrient sensing [258, 259]. Impaired ciliogenesis manifests in over 30 different syndromes with affected eyes, kidneys, brains, and other organs, collectively referred to as ciliopathies [260].

Endocytic regulators have been directly connected to primary ciliogenesis, including Rab8 and Rab11 [261, 262, 263, 264]. Rabin8, a Rab8 guanine nucleotide exchange factor (GEF), is delivered by Rab11 to the centrosome to activate Rab8, which is required for ciliation [261, 262]. Based on interactions with the Rab8 effector MICAL‐L1, significant roles for EHD1 and EHD3 in ciliogenesis were identified [3]. EHD1 knockdown impaired PC formation in RPE‐1 cells, and both EHD1 and EHD3 knockdown impaired PC biogenesis in IMCD3 cells [3]. Moreover, EHD1 and EHD3 are both localized to the ciliary pocket membrane (CPM) [3]. EHD1 also colocalized with Rabin8‐positive preciliary vesicles, but since it associates with the m‐centriole even after Rab8 depletion, it likely functions upstream of the Rab8‐Rab11 cascade during ciliogenesis [3].

Early in ciliogenesis, Myo‐Va–associated preciliary vesicles dock at the distal appendages of the m‐centriole, where they later fuse to generate the ciliary vesicle [262, 265]. For fusion to occur, the centriolar capping protein CP110 must be removed from the m‐centriole [266], allowing axoneme extension and formation of the ciliary membrane [267, 268]. In the absence of EHD1, CP110 was retained on the m‐centriole during serum starvation, thus preventing ciliogenesis [3, 266].

The endosomal scaffold protein, MICAL‐L1, is also required for CP110 removal from the m‐centriole during ciliogenesis; intriguingly, it is required both for the recruitment of EHD1 to tubular recycling endosomes and to the m‐centriole [4]. One mechanism for CP110 removal is that EHD1 is required for the delivery of the E3 ligase HECT domain and RCC1‐like domain 2 (HERC2) on centriolar satellites to the m‐centriole, where the latter ubiquitinates CP110 and promotes its degradation. Indeed, depletion of HERC2 significantly reduced CP110 ubiquitination [269, 270], and EHD1 knockdown significantly decreased the interaction between HERC2 and CP110 [269]. How EHD1 regulates the movement of centriolar satellites remains an outstanding question, but may stem from its ability to regulate microtubule growth [271].

EHD1 may play additional roles in primary ciliogenesis. The EHD1‐interaction protein SNAP29 is a SNARE protein that mediates the fusion of membranes [69]. SNAP29 co‐localized with EHD1 to the ciliary pocket and DAVs, and its recruitment was EHD1‐dependent [3]. Another study showed that the EHD1‐SNAP29 interaction was dependent on EHD1 ATP binding and that expression of a GFP‐EHD1 mutant incapable of binding ATP or SNAP29 was unable to rescue ciliogenesis in EHD1‐depleted cells [107]. These findings support a model that EHD1 mediates SNAP29 recruitment to the mother centriole to promote the fusion of DAVs to form the ciliary vesicle during ciliogenesis [3].

Another EHD1‐interaction partner involved in PC regulation is Syndapin1, as knockdown of the latter impairs ciliogenesis [272]. Syndapin1 localizes with EHD1 on preciliary vesicles at the CPM, and both are present on membrane tubules extending from the CPM and m‐centriole [272]. Knockdown of either Syndapin1 or EHD1 significantly reduced the number of tubules extending from the m‐centriole, suggesting an additional role for EHD1 in ciliogenesis post CP110 cap removal [272]. Taken together, at least three distinct steps have been proposed for EHD1 in ciliogenesis: (1) interaction with MICAL‐L1 to promote HERC2 delivery for CP110 ubiquitination and degradation, (2) SNAP29‐mediated DAV fusion to form the ciliary vesicle, and (3) cooperation with Syndapin1 to promote tubulation of the ciliary vesicle and facilitate PM fusion.

EHD4 has also been implicated in primary ciliogenesis, potentially through its hetero‐oligomerization with EHD1 and EHD3 [55, 63, 107, 205]. To date, EHD2 is the only paralog that has not been implicated in ciliogenesis.

Intriguingly, EHD1 was first described as a candidate gene for Bardet‐Biedl syndrome (BBS1) [2], suggesting its potential involvement in ciliogenesis and ciliopathies. Now, over a quarter century later, the first human disease implicating a single amino acid substitution in the EHD1 protein has been identified in a polycystic kidney disease with features consistent with a ciliopathy [5]. Overall, these findings and the direct connection to ciliopathies support the notion that EHD proteins are not only key regulators of primary ciliogenesis but also play a wider role in cellular function than originally envisioned.

### 7.4 Centrosome Duplication/Cell Division

The involvement of EHD proteins in primary ciliogenesis suggests that they may also regulate additional cellular events. PC appears during the G1‐phase of the cell cycle, and ciliated cells are generally unable to progress through division. However, centrosome duplication occurs during S‐phase, which is required for cell division. Centriole engagement refers to the tight, orthogonal association of parent and progeny centrioles, allowing duplication only once [273, 274]. Upon exit from M‐phase, the centrioles in the newly segregated centrosomes lose their tight association in a process termed “disengagement” [274, 275]. It was previously shown that pericentrin (PCNT) cleavage and CEP215 removal are required for centriole disengagement during cell division [276, 277].

The function of endocytic proteins in the cell cycle has not been extensively studied [278]. However, several studies have shown a role for endocytic proteins in centrosome regulation [265, 279, 280, 281]. EHD1 depletion impaired centriole duplication in U2OS cells and reduced the percentage of disengaged centrioles from 65% in mock‐treated cells to less than 20% [282]. Upon EHD1 depletion, PCNT and CEP215 remained on the centrosome, with CEP215 absent from the spindle midbody [282, 283]. This is likely because EHD1 is required for the transport of CEP215‐containing vesicles away from the centrosome, a step necessary for centriole disengagement [282].

Consistent with a role in the earlier stages of centrosome duplication, EHD1 has also been implicated in cytokinesis. Indeed, EHD1 knockdown led to a significant increase in bi‐ and multi‐nucleated HeLa cells, reaffirming a dysregulated cell cycle [283]. EHD1‐depleted cells displayed both asymmetric cell spreading and cytokinesis failure [283]. EHD1 knockdown also impaired the trafficking of recycling vesicles to the intercellular bridge during cytokinesis, suggesting a role for EHD1 in both cytokinesis and pre‐cytokinetic events [282, 283].

### 7.5 Mitochondrial Homeostasis

EHD proteins have also been linked to mitochondrial function. Mitochondrial homeostasis, key to proper mitochondrial function, relies on continuous cycles of fission and fusion [284, 285]. Dysregulated mitochondrial dynamics manifest in neurological and neurodegenerative diseases, including Parkinson's and Huntington's disease [286, 287, 288]. A role for endosomal proteins in mitochondrial dynamics was demonstrated by overexpression of the retromer component VPS35, which led to shorter, fragmented mitochondria, whereas inhibition of VPS35 elicited longer, more stable mitochondria [289, 290]. The mechanism of VPS35‐mediated mitochondrial fission likely involves lysosomal transport of inactive dynamin‐related protein 1 (Drp1), a GTPase implicated in mitochondrial fission [289, 291, 292].

VPS35 is found in a complex with EHD1 [208]. Similar to VPS35 inhibition, EHD1 depletion led to elongated and more stable mitochondria [293]. EHD1's impact on mitochondrial fission is likely via VPS35; EHD1 depletion reduces VPS35 expression, favoring a model by which it cooperates with VPS35 and the retromer to facilitate Drp1 recycling from the mitochondrial membrane to promote inactive Drp1 turnover and mitochondrial fission [289, 293].

EHD2 has also been connected indirectly with mitochondrial function. Prohibitin (PHB‐1), a mitochondrial protein involved in mitochondrial biogenesis, mitophagy, and regulation of aerobic respiration, was identified as an EHD2 interaction partner [76, 294, 295]. Both EHD2 and PHB localize to lipid droplets (LDs) upon stimulation of lipolysis [81], and it was proposed that EHD2 might regulate PHB‐1 trafficking to mitochondria [76].

### 7.6 Lipid Droplet Dynamics

Lipid droplets (LDs) store fatty acids and neutral lipids and are important for maintenance of energy homeostasis. EHD2 was the first EHD paralog identified on LDs under both steady‐state conditions and upon lipolytic stimulation, suggesting that its localization is independent of lipid droplet structural reorganization during lipolysis [81]. It was later demonstrated that EHD2 localization to LDs relies on the presence of caveolin 1 (Cav1), highlighting an association between LDs and caveolae [74, 296]. Upon stimulation of lipolysis, the density of caveolae on the PM decreases [75], and EHD2 mRNA and protein levels decrease and coincide with reduced LD size [75]. Another study found that during mechano‐adaptation of adipocytes upon lipid loading, EHD2 transfer from caveolae to LDs is dependent on Cav1 phosphorylation [74]. This work highlights mechanical crosstalk between caveolae and LDs, allowing adipocytes to adapt to highly dynamic cellular energy states.

EHD2 mediates the size of LDs by regulating fatty acid uptake through caveolae. While EHD2 knockout mice display fewer LDs per cell in brown adipocytes, they have significantly larger LDs in these cells, likely due to enhanced fatty acid uptake [80]. EHD2 regulation of fatty acid uptake and LD size is dependent on membrane association, oligomerization, and ATPase activity [80], which also controls surface‐associated LDs and their lipolytic activity [73]. EHD1 has also been localized to LDs in mouse embryonic fibroblasts. Indeed, cells derived from EHD1 knockout mice had reduced levels of both total and free cholesterol, potentially as a result of decreased uptake of LDL particles resulting from EHD1 regulation of LDL receptor recycling [33].

Lipophagy is a macroautophagic process for LD degradation; an LD is enveloped in an autophagophore, leading to free fatty acid release [297]. During stress‐induced lipophagy, Rab10 is activated and localized to LC3‐positive autophagic membranes. Activated Rab10 recruits EHBP1 and EHD2 to autophagic membranes, and this trimeric complex promotes autophagic membrane extension during lipophagy, required for LD engulfment [83]. Depletion of Rab10, EHBP1, or EHD2 significantly impaired LD catabolism [83]. EHD2 likely participates by deforming and remodeling the autophagic membrane, but further studies into its mechanism of action during lipophagy are required.

## 8 Mechanisms of EHD Function

### 8.1 EHD Structure and Homology

The N‐terminus of Eps15 has three non‐identical repeats of ~100 residues, which were termed EH domains [298, 299]. EH domains are highly homologous and contain two EF‐hand helix‐loop‐helix motifs connected by an antiparallel β‐sheet [300]. These domains tightly bind asparagine–proline–phenylalanine (NPF) motifs [301, 302]. Using nuclear magnetic resonance (NMR) studies, it was shown that EH domain‐NPF binding is mediated by a conserved hydrophobic pocket of the EH domain and binding is stabilized by hydrophobic and electrostatic interactions [60, 303, 304] (see Figure 1B). Since the initial characterization of the EH domain, over 50 other proteins containing EH‐domains have been identified, including Eps15R, intersectin‐1 and intersectin‐2, REPS1, and others [305]. Many of the EH domain‐containing proteins are involved in endocytosis and endocytic trafficking pathways [306].

Of the EH‐domain‐containing proteins, there are only four highly homologous mammalian paralogs in which the EH domain is localized to the C‐terminus [1, 307] (Figure 1A). Among these paralogs, EHD1 and EHD3 display 86.5% identity at the amino acid level, and the weakest identity is between EHD2 and EHD4, at 67.9% [60, 307] (Figure 1C). These proteins are also characterized by an ATP‐binding G‐domain (Figure 1B) [47, 60, 63, 252]. The two helical regions come together to form a helical domain, which mediates lipid binding [252]. The ATP‐binding G‐domain mediates nucleotide binding and hydrolysis and is homologous to the GTP‐binding domain of Dynamin [192, 252]. In addition to nucleotide binding, the G‐domain also mediates oligomerization. While all EHDs can homo‐oligomerize, EHD1 hetero‐oligomerizes with EHD3 and EHD4; EHD3 preferentially hetero‐oligomerizes with EHD1 but can also interact with EHD4; and EHD4 shows a higher propensity to hetero‐oligomerize with EHD1 [47, 203, 206]. EHD2, the least homologous paralog, favors homo‐oligomerization via a hydrophobic interface of the G‐domain, and mutations within the G‐domain impair dimerization [252, 308]. Mutations within the EHD1 G‐domain impair its ability to both homo‐ and hetero‐oligomerize with both EHD3 and EHD4, and loss of nucleotide binding abrogates EHD1/EHD3 hetero‐ and homo‐oligomerization [47, 203]. While EHD oligomerization is EH domain‐independent, the EH domain mediates interactions with other proteins (Table 2).

**TABLE 2:** EHD protein interaction partners.

EHD paralog	Interacting protein	Interaction region	Interaction region (EHD1)	Biological function	Experimental method	References
EHD1	MICAL‐L1	NPF motif (1)	EH domain	Receptor recycling—endosome fission; EHD1 recruitment to primary cilium	LC‐MS/MS; Y2H; GST pull‐down	[309, 310]
	Rabenosyn‐5	NPF motif (1+2)	EH domain	Receptor recycling	LC‐MS/MS; GST pull‐down	[10]
	Rab11‐FIP2	NPF motif (2)	EH domain	Receptor recycling from the perinuclear endocytic recycling compartment	Y2H	[47]
	EHD1	Coiled‐coil region	Coiled‐coil region	Protein localization and receptor recycling	Y2H	[63, 205]
	EHD3	Coiled‐coil region	Coiled‐coil region	Protein localization and receptor recycling	Y2H; Co‐IP	[205]
	SNAP29	NPF motif	EH domain	Receptor recycling, IGF‐1R receptor internalization	Co‐IP; GST pull‐down	[69, 311]
	Syndapin I	NPF motif	EH domain	Receptor recycling	Co‐IP; GST pull‐down	[312]
	Syndapin II	NPF motif	EH domain	Receptor recycling	Co‐IP; GST pull‐down	[311, 312, 313]
	Snapin	n/a	EH domain	Negatively regulates exocytosis by preventing binding to SNAP25	Co‐IP	[92]
	Fer‐1‐like‐5 (Fer1L5)	NPF motif	EH domain	Myoblast fusion + recycling + muscle repair	Co‐IP; GST pull‐down and radioactive labeling	[30]
	Rabankyrin‐5	NPF motif	EH domain	Retrograde trafficking	Y2H; GST pull‐down; Co‐IP	[208]
	Epsin 1	NPF motif	EH domain	Endocytosis	GST pull‐down	[314]
	Epsin 3	NPF motif	EH domain	Endocytosis	GST pull‐down	[314]
	Cep215	n/a	n/a	Centrosome duplication	Co‐IP	[282]
	Phostensin	64‐ILV(XXXX)LRLS‐75	n/a (EH domain‐independent)	Connects EHD1 and actin during recycling	GST pull‐down	[142]
	Cx43	n/a	n/a	Cardiac remodeling in ischemia	Co‐IP	[24]
	CD44	n/a	n/a	Promotes cancer stemness	Co‐IP	[25, 26]
	Triad1	n/a	EH domain	Neurite growth during spinal cord injury	Co‐IP	[91]
	Insulin‐like growth factor 1 receptor	n/a	n/a	Localization on endocytic vesicles	EHD1 affinity column purification	[69]
	AP‐2 α‐adaptin	n/a	n/a	Localization on endocytic vesicles	EHD1 affinity column purification	[69]
	Clathrin heavy chain	n/a	n/a	Localization on endocytic vesicles	EHD1 affinity column purification	[69]
	Vps35/Vps26/Vps29 complex	n/a	n/a (EH domain‐independent)	Retrograde trafficking	Co‐IP	[315]
	KCa2.3	n/a	n/a	Receptor recycling	Co‐IP	[17]
	Kazrin C	C‐terminal	n/a	Receptor recycling (EE‐ERC)	GST pull‐down	[316]
	cPLA2	n/a	n/a	Receptor recycling (endosome fission)	Co‐IP	[12]
	Amphiphysin	NPF (1) and (2)	EH domain	Receptor recycling	Y2H; GST pull‐down	[192]
	Numb	NPF motif (C‐terminal domain)	EH domain	Clathrin‐independent receptor, Tac recycling impaired in Numb siRNA KD cells (CHO)	Co‐IP	[184]
	Sortilin	n/a	n/a	Sortilin stabilized in macrophages by EHD1 interaction	Co‐IP	[28]
	Programmed death‐ligand 1 (PDL1)	n/a	n/a	Immune evasion in lung adenocarcinoma (A549) cells	Co‐IP	[146]
EHD2	Myoferlin	NPF motif	EH domain	Myoblast fusion + recycling + muscle repair	Co‐IP; GST pull‐down + radioactive labeling	[39, 40]
	Fer‐1‐like‐5 (Fer1L5)	NPF motif	EH domain	Myoblast fusion + recycling + muscle repair	Co‐IP; GST pull‐down + radioactive labeling	[30]
	Prohibitin	n/a	n/a	Regulation of mitochondrial metabolism	Protein crosslink and MALDI‐TOF	[76]
	Rabenosyn‐5	NPF motif	EH domain	Not explored	Y2H	[10]
	EHBP1	NPF motif	EH domain	Actin regulation during endocytosis	GST pull‐down; MALDI‐TOF	[36]
	Epsin 1	NPF motif	EH domain	Endocytosis	GST pull‐down	[314]
	Epsin 3	NPF motif	EH domain	Endocytosis	GST pull‐down	[314]
	Nek3	Coiled‐coil domain	n/a	Modulation of Rac1 activity	Y2H; Co‐IP	[317]
	GLUT4	n/a	n/a	GLUT4 PM trafficking	Co‐IP	[70]
	AP‐1 μ1	n/a	n/a	Not explored	Co‐IP	[70]
	AP‐2 μ2	n/a	n/a	Not explored	Co‐IP	[70]
	CALM	n/a	n/a	Not explored	Co‐IP	[70]
	Caveolin‐1	n/a	n/a	Caveolae stability	Co‐IP	[239]
	Cavin‐1	n/a	n/a	EHD2‐caveolae assembly	GST pull‐down	[239]
EHD3	Rabenosyn‐5	NPF motif	EH domain	Receptor recycling	Y2H	[10]
	Ankyrin‐B	Membrane‐binding domain	Coiled‐coil domain	Stabilizes Na/Ca channels at PM in cardiomyocytes for conductance	GST pull‐downs	[52, 102]
	MICAL‐L1	NPF motif	EH domain	Receptor recycling	Y2H	[309]
	Rab11‐FIP2	NPF motif (2)	EH domain	Receptor recycling from the perinuclear endocytic recycling compartment	Y2H	[47]
	Syndapin I	NPF motif	EH domain	Receptor recycling	GST pull‐downs	[312]
	Syndapin II	NPF motif	EH domain	Receptor recycling	GST pull‐downs	[312]
	Kazrin C	C‐terminal	n/a	Receptor recycling (EE‐ERC)	GST pull‐down	[316]
EHD4	Numb	NPF motif (C‐terminal domain)	EH domain	Clathrin‐independent receptor, Tac recycling impaired in Numb siRNA KD cells (CHO)	Co‐IP; GST pull‐down	[184]
a	Cadherin 23	n/a (non‐NPF motif)	EH domain	EHD4 and CDH23 (protein involved mechanotransduction in Cochlear hair cells) interact	Y2H; Co‐IP	[109]
	Phostensin	64‐ILV(XXXX)LRLS‐75	n/a (EH domain‐independent)	Connects EHD4 and actin during recycling	GST pull‐down	[142]
	EHD1	Helical domain	Helical domain	Endosome fission	Y2H	[203]
	Rabenosyn 5	NPF	EH domain	EHD‐endosome localization; receptor recycling	Y2H	[203]
	Syndapin II	NPF	EH domain	EHD‐endosome localization	Y2H	[203]
	Syndapin I	NPF	EH domain	Receptor recycling	GST pull‐down	[312]
	Type VI collagen	n/a	n/a	Not explored	Type VI collagen affinity blot	[103]

### 8.2 C‐Terminal EH‐Domain Binding

All EH domains interact with proteins containing the tripeptide NPF motif. However, the C‐terminal EHDs display selectivity compared to the rest of the EH‐domain‐containing proteins due to the highly positively charged surface area of the C‐terminal EHDs [60]. For example, the EH domain of EHD1 has a more positively charged surface potential than the EH‐2 domain of Eps15, highlighting a preference for binding to NPF motifs flanked by acidic residues, which form salt‐bridges with the EH domain [309, 310, 318, 319].

For example, the endosomal scaffold protein MICAL‐L1 is a crucial EHD1 interaction partner that contains two NPF motifs, with only the first flanked by acidic residues (NPFEEEEED) and required for EHD1 binding [309, 310]. Additional EHD1‐binding partners that are Rab effectors and have acidic clusters following their NPF motifs include Rabenosyn‐5, Rab11‐FIP2, and Rabankyrin‐5 [10, 47, 208]. In addition to NPF motifs, the EH domain of EHD1 can bind DPF and GPF motifs, and the EH domain of EHD2 can bind GPF motifs, but both bind at a lower affinity than NPF motifs [252, 320].

EHD protein–protein interactions are also mediated through EH domain‐independent mechanisms. For example, EHD1 and EHD4 bind phostensin independently of their EH domains [140, 293], and the worm EHD paralog Rme‐1 can bind to ALX‐1 both via its NPF motif and independently of EH‐NPF interactions [189].

### 8.3 Intracellular Localization and Membrane Association

Despite the sequence homology, the four mammalian EHD paralogs localize to distinct intracellular locations (Figure 2). EHD1 and EHD3 localize to tubular recycling endosomes and Rab11‐positive endosomes containing internalized transferrin and MHC‐I proteins [1, 7, 8, 321]. EHD2 is found at the PM with caveolae, and EHD4 is primarily localized to EEA1‐ and Rab5‐positive early endosomes [55, 321]. EHD1, EHD3, and EHD4 also localize to the primary cilium [3, 4, 107].

A major feature of the endocytic trafficking pathway is the establishment of compartmental identity, which is regulated by local phosphoinositide levels [322]. EHD proteins are capable of directly binding to a variety of phosphatidylinositol moieties as well as phosphatidylserine in a mechanism more dependent on phospholipid charge than structure [192, 252, 321, 323, 324, 325, 326].

The helical domains of EHDs mediate phospholipid binding, and mutation of residues in the central helical region significantly reduces EHD2 association with liposomes in vitro and causes a cytosolic redistribution in cells [252]. Expression of EHD1–4 mutants with abrogated nucleotide binding in cells impairs their membrane association and leads to cytosolic distribution, demonstrating that EHD membrane association is mediated through nucleotide binding [7, 8, 47, 252, 321]. Indeed, EHD2 binding to a lipid monolayer was enhanced when EHD2 was preincubated with the nonhydrolyzable ATP analog AMP‐PNP, supporting the role of ATP binding for membrane association [77]. EHD2 association with membranes is regulated by its ATP cycle: ATP‐bound EHD2 binds to membranes and oligomerizes, and upon ATP hydrolysis there is oligomer disassembly and membrane release [77]. Interestingly, EHD2‐membrane binding stimulates an eightfold increase in its ATPase activity, and EHD1‐membrane binding increases its ATPase activity almost fourfold [252, 326].

Membrane curvature is another important aspect of EHD‐membrane binding. EHD2 selectively binds to smaller liposomes with higher curvature, and EHD1 is preferentially retained on membrane tubules over flat, lipid bilayers [252, 326]. This is in line with the cellular localization of EHD2 to the curved caveolar necks and EHD1 localization to tubular recycling endosomes. Hetero‐oligomerization also regulates membrane binding, as shown by a redistribution of EHD1 in cells upon either EHD4 or EHD1 knockdown [55]. Finally, NMR studies demonstrated that the EH domains of EHD1 and EHD4 directly bind phosphoinositides, suggesting the EH domain is also involved in EHD localization and membrane association [323]. Consistent with these in vitro data, introduction of EHD1‐4 with EH domain deletions into cells disrupted localization to membranes [47, 206, 252, 321, 323]. These findings suggest that EHD protein localization within cells is regulated, at least in part, by their EH domains.

### 8.4 Membrane Fission

EHD1 contains a dynamin‐like nucleotide‐binding domain that binds and hydrolyzes ATP (242, 301). Although early work suggested possible GTP binding based on limited homology to H‐Ras [8], subsequent structural and biochemical studies established that the EHD G‐domain more closely resembles that of dynamin‐like ATPases [192, 252]. From the crystal structure of the EHD2 dimer, the dimerized helical domains adopt a “scissor shape” that binds membranes [252]. EHD‐induced membrane remodeling was first seen when EHD2 was incubated with liposomes, causing significant tubulation [252, 327]. Both the worm RME‐1 protein and the EHD1 *Lampetra fluviatilis* homolog tubulated liposomes in vitro [95, 192]. Moreover, EHD1 incubation with liposomes in the presence of ATP induced liposome vesiculation [204]. In addition, studies in cells demonstrated that EHD1 vesiculates tubular recycling endosomes [12, 328]. Consistent with the delayed recycling of a variety of cargo in EHD1‐depleted cells [7, 8, 9, 11], EHD1 is required for the fission of both canonical and tubular recycling endosomes.

Recent in vitro studies have shed new light on the mechanism of EHD1 involvement in membrane fission. When purified EHD1 was incubated with lipid tubules that extended from a flat, supported lipid bilayer, ATP‐bound EHD1 oligomerized to negatively charged regions, causing membrane sequestration and bulging, leaving the intervening regions of membrane with severe thinning. Ultimately, the membrane tubules thinned to a ~5 nm radius (a critical thickness for spontaneous membrane fission) and underwent fission [326, 329, 330, 331]. This highlights a key mechanistic difference between EHD1‐ and Dynamin1‐mediated membrane fission, as Dynamin1 clusters on membranes, causing membrane thinning in the regions to which it is bound [326]. There was also limited scission of tubules incubated with EHD2 and ATP, but the majority of tubules did not undergo scission, and the ones that did displayed significantly delayed scission, owing to a 40‐fold lower ATPase activity of EHD2 compared to EHD1 [326].

Unlike in vitro studies with purified proteins and liposomes, endosome fission is a complex process in cells that requires the coordination of sequential events by multiple proteins. A current model for endosome fission suggests that the WASH complex is recruited to the neck of budding vesicles on endosomes via the retromer, where it promotes ARP2/3‐mediated branched actin polymerization [332, 333, 334]. During the early stages of endosome fission, actin provides a physical barrier to facilitate cargo sorting and provides a necessary pushing force to generate the appropriate membrane tension required during fission [332, 333, 335, 336, 337]. Negative regulators of actin polymerization, including Coronin 1C [338, 339] and Coronin 2A [340], are recruited during later stages to promote disassembly of the actin network. Accordingly, this facilitates EHD1 recruitment by MICAL‐L1 to the neck of the budding vesicle where it promotes fission (Figure 5) [309, 326, 335, 337, 338, 339, 340, 341, 342].

### 8.5 The Next Quarter Century of EHDs and Outstanding Questions

Despite significant progress in defining the cellular functions of EHD proteins, several fundamental questions remain unresolved. At a mechanistic level, it is still unclear how EHD‐mediated membrane fission is spatially and temporally regulated within cells, and how ATP binding and hydrolysis are coordinated with membrane remodeling. In particular, how EHD proteins coordinate fission events with the actin and microtubule cytoskeleton is largely unknown, although EHD1 binds tubulin and regulates microtubules [271]. Furthermore, whether EHD proteins act autonomously or in concert with additional fission machinery proteins, and how these activities are modulated by lipid composition and post‐translational modifications such as phosphorylation and ubiquitination, remain important areas for investigation.

Another major challenge is understanding functional specificity and redundancy among mammalian EHD paralogs. Although EHD1 and EHD3 share high sequence similarity, they appear to fulfill non‐overlapping roles in certain contexts. How paralog‐specific interactions, expression patterns, and regulatory mechanisms contribute to these differences is not yet well defined. In parallel, the extent to which findings from invertebrate models with a single EHD ortholog can be extrapolated to mammalian systems requires further clarification.

From a physiological and clinical perspective, the recent identification of human disease linked to EHD1 mutations highlights the need to systematically explore the contribution of EHD proteins to tissue‐specific functions and pathologies. Whether additional EHD variants underlie unexplained ciliopathies, kidney disorders, or neurodevelopmental phenotypes remains an open question. Finally, emerging links between EHD proteins and processes such as centrosome dynamics, mitochondrial homeostasis, and cytoskeletal regulation suggest that their functional repertoire may extend well beyond endocytic trafficking, warranting broader investigation.

Addressing these questions will require integrated approaches combining classic biochemistry, structural biology, advanced imaging, genetic models, and human genomics. Together, such efforts promise to refine our understanding of EHD protein function and reveal new principles governing membrane dynamics in health and disease.

## Funding

This work was supported by National Institute of General Medical Sciences (Grant R35GM144102) and the NIH Cancer Biology Training Program (Grant CA009476).

## Ethics Statement

This article is a review article and does not contain any new studies with human participants or animals performed by any of the authors.

## Conflicts of Interest

The authors declare no conflicts of interest.

## Data Availability

Data sharing not applicable to this article as no datasets were generated or analyzed during the current study.

## References

[1] L. Mintz , E. Galperin , M. Pasmanik‐Chor , et al., “EHD1—An EH‐Domain‐Containing Protein With a Specific Expression Pattern,” Genomics 59, no. 1 (1999): 66–76. 10395801 10.1006/geno.1999.5800

[2] N. B. Haider , C. Searby , E. Galperin , et al., “Evaluation and Molecular Characterization of EHD1, a Candidate Gene for Bardet‐Biedl Syndrome 1 (BBS1),” Gene 240, no. 1 (1999): 227–232. 10564830 10.1016/s0378-1119(99)00395-9

[3] Q. Lu , C. Insinna , C. Ott , et al., “Early Steps in Primary Cilium Assembly Require EHD1/EHD3‐Dependent Ciliary Vesicle Formation,” Nature Cell Biology 17, no. 3 (2015): 228–240. 25686250 10.1038/ncb3109 PMC4344897

[4] S. Xie , T. Farmer , N. Naslavsky , and S. Caplan , “MICAL‐L1 Coordinates Ciliogenesis by Recruiting EHD1 to the Primary Cilium,” Journal of Cell Science 132, no. 22 (2019). 10.1242/jcs.233973 PMC6899013 31615969

[5] N. Issler , S. Afonso , I. Weissman , et al., “A Founder Mutation in EHD1 Presents With Tubular Proteinuria and Deafness,” Journal of the American Society of Nephrology 33, no. 4 (2022): 732–745. 35149593 10.1681/ASN.2021101312 PMC8970462

[6] B. Grant , Y. Zhang , M. C. Paupard , S. X. Lin , D. H. Hall , and D. Hirsh , “Evidence That RME‐1, a Conserved *C. elegans* EH‐Domain Protein, Functions in Endocytic Recycling,” Nature Cell Biology 3, no. 6 (2001): 573–579. 11389442 10.1038/35078549

[7] S. X. Lin , B. Grant , D. Hirsh , and F. R. Maxfield , “Rme‐1 Regulates the Distribution and Function of the Endocytic Recycling Compartment in Mammalian Cells,” Nature Cell Biology 3, no. 6 (2001): 567–572. 11389441 10.1038/35078543

[8] S. Caplan , N. Naslavsky , L. M. Hartnell , et al., “A Tubular EHD1‐Containing Compartment Involved in the Recycling of Major Histocompatibility Complex Class I Molecules to the Plasma Membrane,” EMBO Journal 21, no. 11 (2002): 2557–2567. 12032069 10.1093/emboj/21.11.2557 PMC126039

[9] D. Rapaport , W. Auerbach , N. Naslavsky , et al., “Recycling to the Plasma Membrane Is Delayed in EHD1 Knockout Mice,” Traffic 7, no. 1 (2006): 52–60. 16445686 10.1111/j.1600-0854.2005.00359.x

[10] N. Naslavsky , M. Boehm , P. S. Backlund, Jr. , and S. Caplan , “Rabenosyn‐5 and EHD1 Interact and Sequentially Regulate Protein Recycling to the Plasma Membrane,” Molecular Biology of the Cell 15, no. 5 (2004): 2410–2422. 15020713 10.1091/mbc.E03-10-0733 PMC404033

[11] M. Jovic , N. Naslavsky , D. Rapaport , M. Horowitz , and S. Caplan , “EHD1 Regulates Beta1 Integrin Endosomal Transport: Effects on Focal Adhesions, Cell Spreading and Migration,” Journal of Cell Science 120, no. Pt 5 (2007): 802–814. 17284518 10.1242/jcs.03383

[12] B. Cai , S. Caplan , and N. Naslavsky , “cPLA2α and EHD1 Interact and Regulate the Vesiculation of Cholesterol‐Rich, GPI‐Anchored, Protein‐Containing Endosomes,” Molecular Biology of the Cell 23, no. 10 (2012): 1874–1888. 22456504 10.1091/mbc.E11-10-0881 PMC3350552

[13] E. Walseng , O. Bakke , and P. A. Roche , “Major Histocompatibility Complex Class II‐Peptide Complexes Internalize Using a Clathrin‐ and Dynamin‐Independent Endocytosis Pathway,” Journal of Biological Chemistry 283, no. 21 (2008): 14717–14727. 18378669 10.1074/jbc.M801070200 PMC2386912

[14] M. Park , E. C. Penick , J. G. Edwards , J. A. Kauer , and M. D. Ehlers , “Recycling Endosomes Supply AMPA Receptors for LTP,” Science 305, no. 5692 (2004): 1972–1975. 15448273 10.1126/science.1102026

[15] J. A. Picciano , N. Ameen , B. D. Grant , and N. A. Bradbury , “Rme‐1 Regulates the Recycling of the Cystic Fibrosis Transmembrane Conductance Regulator,” American Journal of Physiology. Cell Physiology 285, no. 5 (2003): C1009–C1018. 12839834 10.1152/ajpcell.00140.2003

[16] N. Hardel , N. Harmel , G. Zolles , B. Fakler , and N. Klocker , “Recycling Endosomes Supply Cardiac Pacemaker Channels for Regulated Surface Expression,” Cardiovascular Research 79, no. 1 (2008): 52–60. 18326556 10.1093/cvr/cvn062

[17] Y. Gao , C. M. Balut , M. A. Bailey , G. Patino‐Lopez , S. Shaw , and D. C. Devor , “Recycling of the Ca2+‐Activated K+ Channel, KCa2.3, Is Dependent Upon RME‐1, Rab35/EPI64C, and an N‐Terminal Domain,” Journal of Biological Chemistry 285, no. 23 (2010): 17938–17953. 20360009 10.1074/jbc.M109.086553 PMC2878556

[18] H. J. Chung , X. Qian , M. Ehlers , Y. N. Jan , and L. Y. Jan , “Neuronal Activity Regulates Phosphorylation‐Dependent Surface Delivery of G Protein‐Activated Inwardly Rectifying Potassium Channels,” Proceedings of the National Academy of Sciences of the United States of America 106, no. 2 (2009): 629–634. 19118198 10.1073/pnas.0811615106 PMC2613039

[19] K. Dhawan , N. Naslavsky , and S. Caplan , “Sorting Nexin 17 (SNX17) Links Endosomal Sorting to Eps15 Homology Domain Protein 1 (EHD1)‐Mediated Fission Machinery,” Journal of Biological Chemistry 295, no. 12 (2020): 3837–3850. 32041776 10.1074/jbc.RA119.011368 PMC7086038

[20] S. Bhattacharyya , M. A. Rainey , P. Arya , et al., “Corrigendum: Endocytic Recycling Protein EHD1 Regulates Primary Cilia Morphogenesis and SHH Signaling During Neural Tube Development,” Scientific Reports 7 (2017): 42320. 28332491 10.1038/srep42320 PMC5362947

[21] X. Wu , J. Shen , J. Liu , et al., “Increased EHD1 in Trophoblasts Causes RSM by Activating TGFβ Signaling,” Biology of Reproduction 111, no. 6 (2024): 1235–1248. 39012723 10.1093/biolre/ioae110

[22] S. Chakraborty , A. M. Bhat , I. Mushtaq , et al., “EHD1‐Dependent Traffic of IGF‐1 Receptor to the Cell Surface Is Essential for Ewing Sarcoma Tumorigenesis and Metastasis,” Communications Biology 6, no. 1 (2023): 758. 37474760 10.1038/s42003-023-05125-1 PMC10359273

[23] E. C. M. I. Tom , B. C. Mohapatra , H. Luan , et al., “EHD1 and RUSC2 Control Basal Epidermal Growth Factor Receptor Cell Surface Expression and Recycling,” Molecular and Cellular Biology (2020). 10.1128/MCB.00434-19 PMC7076251 31932478

[24] T. Martins‐Marques , S. Catarino , A. Goncalves , et al., “EHD1 Modulates Cx43 Gap Junction Remodeling Associated With Cardiac Diseases,” Circulation Research 126, no. 10 (2020): e97–e113. 32138615 10.1161/CIRCRESAHA.119.316502

[25] Y. Liu , Y. Song , M. Cao , et al., “A Novel EHD1/CD44/Hippo/SP1 Positive Feedback Loop Potentiates Stemness and Metastasis in Lung Adenocarcinoma,” Clinical and Translational Medicine 12, no. 4 (2022): e836. 35485206 10.1002/ctm2.836 PMC9786223

[26] Y. Lu , W. Wang , and S. Tan , “EHD1 Promotes the Cancer Stem Cell (CSC)‐Like Traits of Glioma Cells via Interacting With CD44 and Suppressing CD44 Degradation,” Environmental Toxicology 37, no. 9 (2022): 2259–2268. 35616188 10.1002/tox.23592

[27] L. R. Cypher , T. A. Bielecki , L. Huang , et al., “Corrigendum to CSF‐1 Receptor Signalling Is Governed by Pre‐Requisite EHD1 Mediated Receptor Display on the Macrophage Cell Surface [Cell Signalling 2016 Sep.; 28(9): 1325‐35],” Cellular Signalling 28, no. 12 (2016): 1933. 27510742 10.1016/j.cellsig.2016.07.018 PMC5555217

[28] F. Ma , Y. Liu , Y. Xu , et al., “Macrophage EHD1 Promotes Inflammation and Stabilizes Sortilin to Accelerate Atherosclerosis,” *bioRxiv* (2025). 10.1161/CIRCRESAHA.125.327751 PMC13196443 42165151

[29] C. C. Yap , Z. M. Lasiecka , S. Caplan , and B. Winckler , “Alterations of EHD1/EHD4 Protein Levels Interfere With L1/NgCAM Endocytosis in Neurons and Disrupt Axonal Targeting,” Journal of Neuroscience 30, no. 19 (2010): 6646–6657. 20463227 10.1523/JNEUROSCI.5428-09.2010 PMC2905050

[30] A. D. Posey, Jr. , P. Pytel , K. Gardikiotes , et al., “Endocytic Recycling Proteins EHD1 and EHD2 Interact With Fer‐1‐Like‐5 (Fer1L5) and Mediate Myoblast Fusion,” Journal of Biological Chemistry 286, no. 9 (2011): 7379–7388. 21177873 10.1074/jbc.M110.157222 PMC3044994

[31] J. B. Reinecke , D. Katafiasz , N. Naslavsky , and S. Caplan , “Regulation of Src Trafficking and Activation by the Endocytic Regulatory Proteins MICAL‐L1 and EHD1,” Journal of Cell Science 127, no. Pt 8 (2014): 1684–1698. 24481818 10.1242/jcs.133892 PMC3986674

[32] T. Wang , Y. Xing , Q. Meng , et al., “Mammalian Eps15 Homology Domain 1 Potentiates Angiogenesis of Non‐Small Cell Lung Cancer by Regulating β2AR Signaling,” Journal of Experimental & Clinical Cancer Research 38, no. 1 (2019): 174. 31023336 10.1186/s13046-019-1162-7 PMC6482525

[33] N. Naslavsky , J. Rahajeng , D. Rapaport , M. Horowitz , and S. Caplan , “EHD1 Regulates Cholesterol Homeostasis and Lipid Droplet Storage,” Biochemical and Biophysical Research Communications 357, no. 3 (2007): 792–799. 17451652 10.1016/j.bbrc.2007.04.022 PMC1978283

[34] M. Bar and A. Avni , “EHD2 Inhibits Signaling of Leucine Rich Repeat Receptor‐Like Proteins,” Plant Signaling & Behavior 4, no. 7 (2009): 682–684. 19820301 10.4161/psb.4.7.9078 PMC2710575

[35] M. Bar , S. Schuster , M. Leibman , R. Ezer , and A. Avni , “The Function of EHD2 in Endocytosis and Defense Signaling Is Affected by SUMO,” Plant Molecular Biology 84, no. 4–5 (2014): 509–518. 24154852 10.1007/s11103-013-0148-7

[36] A. Guilherme , N. A. Soriano , P. S. Furcinitti , and M. P. Czech , “Role of EHD1 and EHBP1 in Perinuclear Sorting and Insulin‐Regulated GLUT4 Recycling in 3T3‐L1 Adipocytes,” Journal of Biological Chemistry 279, no. 38 (2004): 40062–40075. 15247266 10.1074/jbc.M401918200

[37] M. Bar , M. Aharon , S. Benjamin , B. Rotblat , M. Horowitz , and A. Avni , “AtEHDs, Novel Arabidopsis EH‐Domain‐Containing Proteins Involved in Endocytosis,” Plant Journal 55, no. 6 (2008): 1025–1038. 10.1111/j.1365-313X.2008.03571.x 18547399

[38] A. M. Webb , C. R. Francis , R. J. Judson , et al., “EHD2 Modulates Dll4 Endocytosis During Blood Vessel Development,” Microcirculation 29, no. 1 (2022): e12740. 34820962 10.1111/micc.12740 PMC9286817

[39] K. R. Doherty , A. Demonbreun , G. Q. Wallace , et al., “The Endocytic Recycling Protein EHD2 Interacts With Myoferlin to Regulate Myoblast Fusion,” Journal of Biological Chemistry 283, no. 29 (2008): 20252–20260. 18502764 10.1074/jbc.M802306200 PMC2459265

[40] A. R. Demonbreun , M. Quattrocelli , D. Y. Barefield , M. V. Allen , K. E. Swanson , and E. M. McNally , “An Actin‐Dependent Annexin Complex Mediates Plasma Membrane Repair in Muscle,” Journal of Cell Biology 213, no. 6 (2016): 705–718. 27298325 10.1083/jcb.201512022 PMC4915191

[41] H. Q. Yang , K. Jana , M. J. Rindler , and W. A. Coetzee , “The Trafficking Protein, EHD2, Positively Regulates Cardiac Sarcolemmal K(ATP) Channel Surface Expression: Role in Cardioprotection,” FASEB Journal 32, no. 3 (2018): 1613–1625. 29133341 10.1096/fj.201700027R PMC5892718

[42] C. Matthaeus , X. Lian , S. Kunz , et al., “eNOS‐NO‐Induced Small Blood Vessel Relaxation Requires EHD2‐Dependent Caveolae Stabilization,” PLoS One 14, no. 10 (2019): e0223620. 31600286 10.1371/journal.pone.0223620 PMC6786623

[43] H. Luan , T. A. Bielecki , B. C. Mohapatra , et al., “EHD2 Overexpression Promotes Tumorigenesis and Metastasis in Triple‐Negative Breast Cancer by Regulating Store‐Operated Calcium Entry,” eLife 12 (2023): e81288. 36625722 10.7554/eLife.81288 PMC9988264

[44] M. Li , X. Yang , J. Zhang , et al., “Effects of EHD2 Interference on Migration of Esophageal Squamous Cell Carcinoma,” Medical Oncology 30, no. 1 (2013): 396. 23354948 10.1007/s12032-012-0396-4 PMC3586404

[45] J. Liu , W. Ni , L. Qu , et al., “Decreased Expression of EHD2 Promotes Tumor Metastasis and Indicates Poor Prognosis in Hepatocellular Carcinoma,” Digestive Diseases and Sciences 61, no. 9 (2016): 2554–2567. 27221498 10.1007/s10620-016-4202-6

[46] Y. Shi , X. Liu , Y. Sun , et al., “Decreased Expression and Prognostic Role of EHD2 in Human Breast Carcinoma: Correlation With E‐Cadherin,” Journal of Molecular Histology 46, no. 2 (2015): 221–231. 25758127 10.1007/s10735-015-9614-7

[47] N. Naslavsky , J. Rahajeng , M. Sharma , M. Jovic , and S. Caplan , “Interactions Between EHD Proteins and Rab11‐FIP2: A Role for EHD3 in Early Endosomal Transport,” Molecular Biology of the Cell 17, no. 1 (2006): 163–177. 16251358 10.1091/mbc.E05-05-0466 PMC1345656

[48] N. Naslavsky , J. McKenzie , N. Altan‐Bonnet , D. Sheff , and S. Caplan , “EHD3 Regulates Early‐Endosome‐To‐Golgi Transport and Preserves Golgi Morphology,” Journal of Cell Science 122, no. Pt 3 (2009): 389–400. 19139087 10.1242/jcs.037051 PMC2724733

[49] J. Curran , H. Musa , C. F. Kline , et al., “Eps15 Homology Domain‐Containing Protein 3 Regulates Cardiac T‐Type Ca2+ Channel Targeting and Function in the Atria,” Journal of Biological Chemistry 290, no. 19 (2015): 12210–12221. 25825486 10.1074/jbc.M115.646893 PMC4424353

[50] M. Amessou , A. S. Ebrahim , A. Dilly , et al., “Spatio‐Temporal Regulation of EGFR Signaling by the Eps15 Homology Domain‐Containing Protein 3 (EHD3),” Oncotarget 7, no. 48 (2016): 79203–79216. 27811356 10.18632/oncotarget.13008 PMC5346708

[51] N. C. Waxmonsky and S. D. Conner , “Αvβ3‐Integrin‐Mediated Adhesion Is Regulated Through an AAK1L‐ and EHD3‐Dependent Rapid‐Recycling Pathway,” Journal of Cell Science 126, no. Pt 16 (2013): 3593–3601. 23781025 10.1242/jcs.122465 PMC3744025

[52] J. Curran , M. A. Makara , S. C. Little , et al., “EHD3‐Dependent Endosome Pathway Regulates Cardiac Membrane Excitability and Physiology,” Circulation Research 115, no. 1 (2014): 68–78. 24759929 10.1161/CIRCRESAHA.115.304149 PMC4065849

[53] A. Joset , D. A. Dodd , S. Halegoua , and M. E. Schwab , “Pincher‐Generated Nogo‐A Endosomes Mediate Growth Cone Collapse and Retrograde Signaling,” Journal of Cell Biology 188, no. 2 (2010): 271–285. 20083601 10.1083/jcb.200906089 PMC2812518

[54] Y. Shao , W. Akmentin , J. J. Toledo‐Aral , et al., “Pincher, a Pinocytic Chaperone for Nerve Growth Factor/TrkA Signaling Endosomes,” Journal of Cell Biology 157, no. 4 (2002): 679–691. 12011113 10.1083/jcb.200201063 PMC2173850

[55] M. Sharma , N. Naslavsky , and S. Caplan , “A Role for EHD4 in the Regulation of Early Endosomal Transport,” Traffic 9, no. 6 (2008): 995–1018. 18331452 10.1111/j.1600-0854.2008.00732.x PMC2795359

[56] T. S. Malinova , A. Angulo‐Urarte , J. Nuchel , et al., “A Junctional PACSIN2/EHD4/MICAL‐L1 Complex Coordinates VE‐Cadherin Trafficking for Endothelial Migration and Angiogenesis,” Nature Communications 12, no. 1 (2021): 2610. 10.1038/s41467-021-22873-y PMC8110786 33972531

[57] W. Dun , P. Danilo, Jr. , P. J. Mohler , and P. A. Boyden , “Microtubular Remodeling and Decreased Expression of Nav1.5 With Enhanced EHD4 in Cells From the Infarcted Heart,” Life Sciences 201 (2018): 72–80. 29534991 10.1016/j.lfs.2018.03.024

[58] F. M. Iseka , B. T. Goetz , I. Mushtaq , et al., “Role of the EHD Family of Endocytic Recycling Regulators for TCR Recycling and T Cell Function,” Journal of Immunology 200, no. 2 (2018): 483–499. 10.4049/jimmunol.1601793 PMC5760286 29212907

[59] B. D. Grant and S. Caplan , “Mechanisms of EHD/RME‐1 Protein Function in Endocytic Transport,” Traffic 9, no. 12 (2008): 2043–2052. 18801062 10.1111/j.1600-0854.2008.00834.x PMC2766864

[60] N. Naslavsky and S. Caplan , “EHD Proteins: Key Conductors of Endocytic Transport,” Trends in Cell Biology 21, no. 2 (2011): 122–131. 21067929 10.1016/j.tcb.2010.10.003 PMC3052690

[61] N. Naslavsky and S. Caplan , “The Enigmatic Endosome – Sorting the Ins and Outs of Endocytic Trafficking,” Journal of Cell Science 131, no. 13 (2018). 10.1242/jcs.216499 PMC6051342 29980602

[62] N. Naslavsky and S. Caplan , “C‐Terminal EH‐Domain‐Containing Proteins: Consensus for a Role in Endocytic Trafficking, EH?,” Journal of Cell Science 118, no. Pt 18 (2005): 4093–4101. 16155252 10.1242/jcs.02595

[63] D. W. Lee , X. Zhao , S. Scarselletta , et al., “ATP Binding Regulates Oligomerization and Endosome Association of RME‐1 Family Proteins,” Journal of Biological Chemistry 280 (2005): 280–290. 10.1074/jbc.M412751200 15710626

[64] H. Gudmundsson , J. Curran , F. Kashef , et al., “Differential Regulation of EHD3 in Human and Mammalian Heart Failure,” Journal of Molecular and Cellular Cardiology 52, no. 5 (2012): 1183–1190. 22406195 10.1016/j.yjmcc.2012.02.008 PMC3360944

[65] D. Tong , Y. N. Liang , A. A. Stepanova , et al., “Increased Eps15 Homology Domain 1 and RAB11FIP3 Expression Regulate Breast Cancer Progression via Promoting Epithelial Growth Factor Receptor Recycling,” Tumour Biology 39, no. 2 (2017): 1010428317691010. 28215104 10.1177/1010428317691010

[66] Y. Liu , Y. Liang , M. Li , et al., “Eps15 Homology Domain 1 Promotes the Evolution of Papillary Thyroid Cancer by Regulating Endocytotic Recycling of Epidermal Growth Factor Receptor,” Oncology Letters 16, no. 4 (2018): 4263–4270. 30214560 10.3892/ol.2018.9200 PMC6126170

[67] J. Huang , F. Tian , Y. Song , et al., “A Feedback Circuit Comprising EHD1 and 14‐3‐3ζ Sustains β‐Catenin/c‐Myc‐Mediated Aerobic Glycolysis and Proliferation in Non‐Small Cell Lung Cancer,” Cancer Letters 520 (2021): 12–25. 34217785 10.1016/j.canlet.2021.06.023

[68] J. Yu , Y. Yan , C. Hua , and H. Song , “EHD3 Promotes Gastric Cancer Progression via Wnt/β‐Catenin/EMT Pathway and Associates With Clinical Prognosis and Immune Infiltration,” American Journal of Cancer Research 13, no. 9 (2023): 4401–4417. 37818061 PMC10560930

[69] R. Rotem‐Yehudar , E. Galperin , and M. Horowitz , “Association of Insulin‐Like Growth Factor 1 Receptor With EHD1 and SNAP29,” Journal of Biological Chemistry 276, no. 35 (2001): 33054–33060. 11423532 10.1074/jbc.M009913200

[70] S. Y. Park , B. G. Ha , G. H. Choi , et al., “EHD2 Interacts With the Insulin‐Responsive Glucose Transporter (GLUT4) in Rat Adipocytes and May Participate in Insulin‐Induced GLUT4 Recruitment,” Biochemistry 43, no. 23 (2004): 7552–7562. 15182197 10.1021/bi049970f

[71] P. Baca , F. Barajas‐Olmos , E. Mirzaeicheshmeh , et al., “DNA Methylation and Gene Expression Analysis in Adipose Tissue to Identify New Loci Associated With T2D Development in Obesity,” Nutrition & Diabetes 12, no. 1 (2022): 50. 36535927 10.1038/s41387-022-00228-w PMC9763387

[72] M. Varela‐Guruceaga , E. Belaidi , L. Lebeau , et al., “Intermittent Hypoxia Mediates Caveolae Disassembly That Parallels Insulin Resistance Development,” Frontiers in Physiology 11 (2020): 565486. 33324235 10.3389/fphys.2020.565486 PMC7726350

[73] B. H. B. Morén , F. Negoita , C. Fryklund , R. Lundmark , O. Göransson , and K. G. Stenkula , “EHD2 Regulates Adipocyte Function and Is Enriched at Cell Surface‐Associated Lipid Droplets in Primary Human Adipocytes,” Molecular Biology of the Cell 30, no. 10 (2019): 1147–1159. 30811273 10.1091/mbc.E18-10-0680 PMC6724522

[74] M. C. M. Aboy‐Pardal , M. C. Guadamillas , C. R. Guerrero , et al., “Plasma Membrane Remodeling Determines Adipocyte Expansion and Mechanical Adaptability,” Nature Communications 15, no. 1 (2024): 10102. 10.1038/s41467-024-54224-y PMC11605069 39609408

[75] N. Briand , C. Prado , G. Mabilleau , et al., “Caveolin‐1 Expression and Cavin Stability Regulate Caveolae Dynamics in Adipocyte Lipid Store Fluctuation,” Diabetes 63, no. 12 (2014): 4032–4044. 24969108 10.2337/db13-1961 PMC4238006

[76] M. Vessal , S. Mishra , S. Moulik , and L. J. Murphy , “Prohibitin Attenuates Insulin‐Stimulated Glucose and Fatty Acid Oxidation in Adipose Tissue by Inhibition of Pyruvate Carboxylase,” FEBS Journal 273, no. 3 (2006): 568–576. 16420480 10.1111/j.1742-4658.2005.05090.x

[77] M. Hoernke , J. Mohan , E. Larsson , et al., “EHD2 Restrains Dynamics of Caveolae by an ATP‐Dependent, Membrane‐Bound, Open Conformation,” Proceedings of the National Academy of Sciences of the United States of America 114, no. 22 (2017): E4360–e4369. 28223496 10.1073/pnas.1614066114 PMC5465919

[78] E. Larsson , B. Morén , K. A. McMahon , R. G. Parton , and R. Lundmark , “Dynamin2 Functions as an Accessory Protein to Reduce the Rate of Caveola Internalization,” Journal of Cell Biology 222, no. 4 (2023): e202205122. 36729022 10.1083/jcb.202205122 PMC9929934

[79] L. C. Simone , N. Naslavsky , and S. Caplan , “Scratching the Surface: Actin' and Other Roles for the C‐Terminal Eps15 Homology Domain Protein, EHD2,” Histology and Histopathology 29, no. 3 (2014): 285–292. 24347515 10.14670/hh-29.285 PMC4106284

[80] C. Matthaeus , I. Lahmann , S. Kunz , et al., “EHD2‐Mediated Restriction of Caveolar Dynamics Regulates Cellular Fatty Acid Uptake,” Proceedings of the National Academy of Sciences of the United States of America 117, no. 13 (2020): 7471–7481. 32170013 10.1073/pnas.1918415117 PMC7132307

[81] D. L. Brasaemle , G. Dolios , L. Shapiro , and R. Wang , “Proteomic Analysis of Proteins Associated With Lipid Droplets of Basal and Lipolytically Stimulated 3T3‐L1 Adipocytes,” Journal of Biological Chemistry 279, no. 45 (2004): 46835–46842. 15337753 10.1074/jbc.M409340200

[82] C. M. Blouin , S. Le Lay , A. Eberl , et al., “Lipid Droplet Analysis in Caveolin‐Deficient Adipocytes: Alterations in Surface Phospholipid Composition and Maturation Defects[S],” Journal of Lipid Research 51, no. 5 (2010): 945–956. 19965594 10.1194/jlr.M001016 PMC2853462

[83] Z. Li , R. J. Schulze , S. G. Weller , et al., “A Novel Rab10‐EHBP1‐EHD2 Complex Essential for the Autophagic Engulfment of Lipid Droplets,” Science Advances 2, no. 12 (2016): e1601470. 28028537 10.1126/sciadv.1601470 PMC5161429

[84] N. Aboulaich , J. P. Vainonen , P. Stralfors , and A. V. Vener , “Vectorial Proteomics Reveal Targeting, Phosphorylation and Specific Fragmentation of Polymerase I and Transcript Release Factor (PTRF) at the Surface of Caveolae in Human Adipocytes,” Biochemical Journal 383, no. Pt 2 (2004): 237–248. 15242332 10.1042/BJ20040647 PMC1134064

[85] C. Bregnard , A. Zamborlini , M. Leduc , et al., “Comparative Proteomic Analysis of HIV‐1 Particles Reveals a Role for Ezrin and EHD4 in the Nef‐Dependent Increase of Virus Infectivity,” Journal of Virology 87, no. 7 (2013): 3729–3740. 23325686 10.1128/JVI.02477-12 PMC3624205

[86] A. Dumas , G. Lê‐Bury , F. Marie‐Anaïs , et al., “The HIV‐1 Protein Vpr Impairs Phagosome Maturation by Controlling Microtubule‐Dependent Trafficking,” Journal of Cell Biology 211, no. 2 (2015): 359–372. 26504171 10.1083/jcb.201503124 PMC4621833

[87] A. S. Dhanda , C. Yu , K. T. Lulic , et al., “ *Listeria monocytogenes* Exploits Host Caveolin for Cell‐to‐Cell Spreading,” MBio 11, no. 1 (2020), 10.1128/mBio.02857-19. PMC6974566 31964732

[88] H. Kobayashi and M. Fukuda , “Rab35 Establishes the EHD1‐Association Site by Coordinating Two Distinct Effectors During Neurite Outgrowth,” Journal of Cell Science 126, no. Pt 11 (2013): 2424–2435. 23572513 10.1242/jcs.117846

[89] H. Kobayashi , K. Etoh , N. Ohbayashi , and M. Fukuda , “Rab35 Promotes the Recruitment of Rab8, Rab13 and Rab36 to Recycling Endosomes Through MICAL‐L1 During Neurite Outgrowth,” Biology Open 3, no. 9 (2014): 803–814. 25086062 10.1242/bio.20148771 PMC4163657

[90] C. Wu , Z. Cui , Y. Liu , et al., “The Importance of EHD1 in Neurite Outgrowth Contributing to the Functional Recovery After Spinal Cord Injury,” International Journal of Developmental Neuroscience 52 (2016): 24–32. 27211346 10.1016/j.ijdevneu.2016.05.007

[91] C. Wu , G. Bao , G. Xu , et al., “Triad1 Regulates the Expression and Distribution of EHD1 Contributing to the Neurite Outgrowth of Neurons After Spinal Cord Injury,” Journal of Cellular Biochemistry 120, no. 4 (2019): 5355–5366. 30320922 10.1002/jcb.27814

[92] S. Wei , Y. Xu , H. Shi , et al., “EHD1 Is a Synaptic Protein That Modulates Exocytosis Through Binding to Snapin,” Molecular and Cellular Neurosciences 45, no. 4 (2010): 418–429. 20696250 10.1016/j.mcn.2010.07.014

[93] K. Ke , Y. Rui , L. Li , et al., “Upregulation of EHD2 After Intracerebral Hemorrhage in Adult Rats,” Journal of Molecular Neuroscience 54, no. 2 (2014): 171–180. 24664435 10.1007/s12031-014-0271-1

[94] S. Yan , Y. Wang , Y. Zhang , et al., “Synaptotagmin‐11 Regulates the Functions of Caveolae and Responds to Mechanical Stimuli in Astrocytes,” FASEB Journal 34, no. 2 (2020): 2609–2624. 31908017 10.1096/fj.201901715R

[95] J. Jakobsson , F. Ackermann , F. Andersson , D. Larhammar , P. Low , and L. Brodin , “Regulation of Synaptic Vesicle Budding and Dynamin Function by an EHD ATPase,” Journal of Neuroscience 31, no. 39 (2011): 13972–13980. 21957258 10.1523/JNEUROSCI.1289-11.2011 PMC6633164

[96] G. Valdez , W. Akmentin , P. Philippidou , R. Kuruvilla , D. D. Ginty , and S. Halegoua , “Pincher‐Mediated Macroendocytosis Underlies Retrograde Signaling by Neurotrophin Receptors,” Journal of Neuroscience 25, no. 21 (2005): 5236–5247. 15917464 10.1523/JNEUROSCI.5104-04.2005 PMC6724820

[97] P. Philippidou , G. Valdez , W. Akmentin , W. J. Bowers , H. J. Federoff , and S. Halegoua , “Trk Retrograde Signaling Requires Persistent, Pincher‐Directed Endosomes,” Proceedings of the National Academy of Sciences of the United States of America 108, no. 2 (2011): 852–857. 21187387 10.1073/pnas.1015981108 PMC3021064

[98] K. Koles , E. M. Messelaar , Z. Feiger , C. J. Yu , C. A. Frank , and A. A. Rodal , “The EHD Protein Past1 Controls Postsynaptic Membrane Elaboration and Synaptic Function,” Molecular Biology of the Cell 26, no. 18 (2015): 3275–3288. 26202464 10.1091/mbc.E15-02-0093 PMC4569317

[99] A. D. Posey, Jr. , K. E. Swanson , M. G. Alvarez , et al., “EHD1 Mediates Vesicle Trafficking Required for Normal Muscle Growth and Transverse Tubule Development,” Developmental Biology 387, no. 2 (2014): 179–190. 24440153 10.1016/j.ydbio.2014.01.004 PMC3987670

[100] A. R. Demonbreun , K. E. Swanson , A. E. Rossi , et al., “Eps 15 Homology Domain (EHD)‐1 Remodels Transverse Tubules in Skeletal Muscle,” PLoS One 10, no. 9 (2015): e0136679. 26325203 10.1371/journal.pone.0136679 PMC4556691

[101] A. Marg , V. Schoewel , T. Timmel , et al., “Sarcolemmal Repair Is a Slow Process and Includes EHD2,” Traffic 13, no. 9 (2012): 1286–1294. 22679923 10.1111/j.1600-0854.2012.01386.x

[102] H. Gudmundsson , T. J. Hund , P. J. Wright , et al., “EH Domain Proteins Regulate Cardiac Membrane Protein Targeting,” Circulation Research 107, no. 1 (2010): 84–95. 20489164 10.1161/CIRCRESAHA.110.216713 PMC2901408

[103] H. J. Kuo , N. T. Tran , S. A. Clary , N. P. Morris , and R. W. Glanville , “Characterization of EHD4, an EH Domain‐Containing Protein Expressed in the Extracellular Matrix,” Journal of Biological Chemistry 276, no. 46 (2001): 43103–43110. 11533061 10.1074/jbc.M106128200

[104] S. T. Khan , N. Ahuja , S. Taïb , S. Vohra , O. Cleaver , and S. S. Nunes , “Single‐Cell Meta‐Analysis Uncovers the Pancreatic Endothelial Cell Transcriptomic Signature and Reveals a Key Role for NKX2‐3 in PLVAP Expression,” Arteriosclerosis, Thrombosis, and Vascular Biology 44, no. 12 (2024): 2596–2615. 39445426 10.1161/ATVBAHA.124.321781 PMC11594071

[105] J. Liu , Y. Li , F. Li , X. Zhang , Y. Wang , and J. Zhou , “Landscape of Extrachromosomal Circular DNAs, Transcriptome, and Proteome Analysis Reveals Insights Into Alcoholic Liver Cirrhosis,” Gene 927 (2024): 148599. 38782221 10.1016/j.gene.2024.148599

[106] A. Desroches‐Castan , E. Tillet , N. Ricard , et al., “Bone Morphogenetic Protein 9 Is a Paracrine Factor Controlling Liver Sinusoidal Endothelial Cell Fenestration and Protecting Against Hepatic Fibrosis,” Hepatology 70, no. 4 (2019): 1392–1408. 30964206 10.1002/hep.30655

[107] T. Jones , N. Naslavsky , and S. Caplan , “Differential Requirements for the Eps15 Homology Domain Proteins EHD4 and EHD2 in the Regulation of Mammalian Ciliogenesis,” Traffic 23, no. 7 (2022): 360–373. 35510564 10.1111/tra.12845 PMC9324998

[108] H. Zhao , Z. Khan , and C. J. Westlake , “Ciliogenesis Membrane Dynamics and Organization,” Seminars in Cell & Developmental Biology 133 (2023): 20–31. 35351373 10.1016/j.semcdb.2022.03.021 PMC9510604

[109] S. Sengupta , M. George , K. K. Miller , et al., “EHD4 and CDH23 Are Interacting Partners in Cochlear Hair Cells,” Journal of Biological Chemistry 284, no. 30 (2009): 20121–20129. 19487694 10.1074/jbc.M109.025668 PMC2740438

[110] K. Meindl , N. Issler , S. Afonso , et al., “A Missense Mutation in Ehd1 Associated With Defective Spermatogenesis and Male Infertility,” Frontiers in Cell and Development Biology 11 (2023): 1240558. 10.3389/fcell.2023.1240558 PMC10600459 37900275

[111] M. Taniwaki , Y. Daigo , N. Ishikawa , et al., “Gene Expression Profiles of Small‐Cell Lung Cancers: Molecular Signatures of Lung Cancer,” International Journal of Oncology 29, no. 3 (2006): 567–575. 16865272

[112] J. C. Desmond , S. Raynaud , E. Tung , W. K. Hofmann , T. Haferlach , and H. P. Koeffler , “Discovery of Epigenetically Silenced Genes in Acute Myeloid Leukemias,” Leukemia 21, no. 5 (2007): 1026–1034. 17330099 10.1038/sj.leu.2404611

[113] Y. H. Wang , S. C. Chang , M. Ansar , C. S. Hung , and R. K. Lin , “Eps15 Homology Domain‐Containing Protein 3 Hypermethylation as a Prognostic and Predictive Marker for Colorectal Cancer,” Biomedicine 9, no. 5 (2021): 453. 10.3390/biomedicines9050453 PMC8145505 33922189

[114] S. Chukkapalli , M. Amessou , H. Dekhil , et al., “Ehd3, a Regulator of Vesicular Trafficking, Is Silenced in Gliomas and Functions as a Tumor Suppressor by Controlling Cell Cycle Arrest and Apoptosis,” Carcinogenesis 35, no. 4 (2014): 877–885. 24306026 10.1093/carcin/bgt399

[115] Y. Chen , L. D. Liao , Z. Y. Wu , et al., “Identification of Key Genes by Integrating DNA Methylation and Next‐Generation Transcriptome Sequencing for Esophageal Squamous Cell Carcinoma,” Aging 12, no. 2 (2020): 1332–1365. 31962291 10.18632/aging.102686 PMC7053602

[116] S. B. Sonne , R. Yadav , G. Yin , et al., “Obesity Is Associated With Depot‐Specific Alterations in Adipocyte DNA Methylation and Gene Expression,” Adipocytes 6, no. 2 (2017): 124–133. 10.1080/21623945.2017.1320002 PMC5477735 28481699

[117] K. C. Moon , J. A. Gim , D. S. Kim , C. W. Choi , J. Yoon , and S. Y. Yoon , “Total Platelet Transcriptomics and Its Network Analysis by RNA‐Seq and miRNA‐Seq and PCA Application in Essential Thrombocythaemia,” Acta Haematologica 144, no. 3 (2021): 337–344. 33227791 10.1159/000510459

[118] D. S. Miotto , F. Duchatsch , A. Dionizio , M. A. R. Buzalaf , and S. L. Amaral , “Physical Training vs. Perindopril Treatment on Arterial Stiffening of Spontaneously Hypertensive Rats: A Proteomic Analysis and Possible Mechanisms,” Biomedicine 11, no. 5 (2023): 1381. 10.3390/biomedicines11051381 PMC10216057 37239052

[119] R. D. S. Francisco Junior , J. R. Temerozo , C. D. S. Ferreira , et al., “Differential Haplotype Expression in Class I MHC Genes During SARS‐CoV‐2 Infection of Human Lung Cell Lines,” Frontiers in Immunology 13 (2022): 1101526. 36818472 10.3389/fimmu.2022.1101526 PMC9929942

[120] Y. H. Zhang , M. R. Hoopmann , P. J. Castaldi , et al., “Lung Proteomic Biomarkers Associated With Chronic Obstructive Pulmonary Disease,” American Journal of Physiology. Lung Cellular and Molecular Physiology 321, no. 6 (2021): L1119–l1130. 34668408 10.1152/ajplung.00198.2021 PMC8715017

[121] J. Lin , Y. Nan , J. Sun , et al., “Identification and Construction of a R‐Loop Mediated Diagnostic Model and Associated Immune Microenvironment of COPD Through Machine Learning and Single‐Cell Transcriptomics,” Inflammation 48, no. 4 (2025): 2802–2823. 39798034 10.1007/s10753-024-02232-x PMC12336087

[122] J. Cheng , D. Ji , Y. Yin , et al., “Proteomic Profiling of Urinary Small Extracellular Vesicles in Children With Pneumonia: A Pilot Study,” Pediatric Research 94, no. 1 (2023): 161–171. 36635400 10.1038/s41390-022-02431-y PMC9838271

[123] K. Xie , Y. Sun , X. Li , et al., “Biomarkers and Pathways in Autism Spectrum Disorder: An Individual Meta‐Analysis Based on Proteomic and Metabolomic Data,” European Archives of Psychiatry and Clinical Neuroscience 275, no. 8 (2025): 2461–2477. 39361099 10.1007/s00406-024-01922-9

[124] C. Shi , K. Zhang , X. Wang , Y. Shen , and Q. Xu , “A Study of the Combined Effects of the EHD3 and FREM3 Genes in Patients With Major Depressive Disorder,” American Journal of Medical Genetics. Part B, Neuropsychiatric Genetics 159B, no. 3 (2012): 336–342. 10.1002/ajmg.b.32033 22337703

[125] L. Wang , C. Shi , K. Zhang , and Q. Xu , “The Gender‐Specific Association of EHD3 Polymorphisms With Major Depressive Disorder,” Neuroscience Letters 567 (2014): 11–14. 24607927 10.1016/j.neulet.2014.02.055

[126] L. Wang , C. J. Shi , and Q. Xu , “Association Between EHD3 Gene and the Cognitive Function of Patients With Major Depressive Disorder,” Zhongguo Yi Xue Ke Xue Yuan Xue Bao. Acta Academiae Medicinae Sinicae 36, no. 3 (2014): 227–233. 24997812 10.3881/j.issn.1000-503X.2014.03.001

[127] N. Kouri , M. E. Murray , J. S. Reddy , et al., “Latent Trait Modeling of Tau Neuropathology in Progressive Supranuclear Palsy,” Acta Neuropathologica 141, no. 5 (2021): 667–680. 33635380 10.1007/s00401-021-02289-0 PMC8043857

[128] D. F. V. Pirscoveanu , D. G. Olaru , D. M. Hermann , T. R. Doeppner , F. S. Ghinea , and A. Popa‐Wagner , “Immune Genes Involved in Synaptic Plasticity During Early Postnatal Brain Development Contribute to Post‐Stroke Damage in the Aging Male Rat Brain,” Biogerontology 26, no. 2 (2025): 60. 39966204 10.1007/s10522-025-10203-4 PMC12021737

[129] D. Stacey , A. Lourdusamy , B. Ruggeri , et al., “A Translational Systems Biology Approach in Both Animals and Humans Identifies a Functionally Related Module of Accumbal Genes Involved in the Regulation of Reward Processing and Binge Drinking in Males,” Journal of Psychiatry & Neuroscience 41, no. 3 (2016): 192–202. 26679926 10.1503/jpn.150138 PMC4853210

[130] E. Evergren , I. G. Mills , and G. Kennedy , “Adaptations of Membrane Trafficking in Cancer and Tumorigenesis,” Journal of Cell Science 137, no. 10 (2024): jcs260943. 38770683 10.1242/jcs.260943 PMC11166456

[131] H. Yu , G. Qu , Y. Wang , et al., “The Expression of Eps15 Homology Domain 1 Is Negatively Correlated With Disease‐Free Survival and Overall Survival of Osteosarcoma Patients,” Journal of Orthopaedic Surgery and Research 14, no. 1 (2019): 103. 30975166 10.1186/s13018-019-1137-6 PMC6460645

[132] P. Decruyenaere , E. Giuili , K. Verniers , et al., “Exploring the Cell‐Free Total RNA Transcriptome in Diffuse Large B‐Cell Lymphoma and Primary Mediastinal B‐Cell Lymphoma Patients as Biomarker Source in Blood Plasma Liquid Biopsies,” Frontiers in Oncology 13 (2023): 1221471. 37954086 10.3389/fonc.2023.1221471 PMC10634215

[133] K. Iżykowska , G. K. Przybylski , C. Gand , et al., “Genetic Rearrangements Result in Altered Gene Expression and Novel Fusion Transcripts in Sézary Syndrome,” Oncotarget 8, no. 24 (2017): 39627–39639. 28489605 10.18632/oncotarget.17383 PMC5503638

[134] J. Gao , Q. Meng , Y. Zhao , X. Chen , and L. Cai , “EHD1 Confers Resistance to Cisplatin in Non‐Small Cell Lung Cancer by Regulating Intracellular Cisplatin Concentrations,” BMC Cancer 16 (2016): 470. 27411790 10.1186/s12885-016-2527-3 PMC4944258

[135] R. Shrestha , N. Nabavi , S. Volik , et al., “Well‐Differentiated Papillary Mesothelioma of the Peritoneum Is Genetically Distinct From Malignant Mesothelioma,” Cancers 12, no. 6 (2020): 1568. 32545767 10.3390/cancers12061568 PMC7352777

[136] Y. Nagao , N. Mimura , J. Takeda , et al., “Genetic and Transcriptional Landscape of Plasma Cells in POEMS Syndrome,” Leukemia 33, no. 7 (2019): 1723–1735. 30635632 10.1038/s41375-018-0348-x

[137] Q. Meng , Y. Xing , T. Ren , et al., “Mammalian Eps15 Homology Domain 1 Promotes Metastasis in Non‐Small Cell Lung Cancer by Inducing Epithelial‐Mesenchymal Transition,” Oncotarget 8, no. 14 (2017): 22433–22442. 27531895 10.18632/oncotarget.11220 PMC5410234

[138] X. Gao , J. Li , X. Feng , et al., “EHD1 Promotes Breast Cancer Metastasis Through Upregulating HIF2a Expression via Activating mTOR Pathway,” FASEB Journal 38, no. 21 (2024): e70168. 39530565 10.1096/fj.202401919R

[139] S. Qiu , L. Ma , K. Yu , et al., “A Pathological Role of O‐GlcNAcylation‐Driven TR11B Production and Function in Lung Adenocarcinoma,” Developmental Cell 60, no. 23 (2025): 3321–3338.e3312. 40930100 10.1016/j.devcel.2025.08.010

[140] Y. S. Lin , K. Y. Huang , H. C. Yu , et al., “Identification of Phostensin in Association With Eps 15 Homology Domain‐Containing Protein 1 (EHD1) and EHD4,” Biochemical and Biophysical Research Communications 531, no. 2 (2020): 236–241. 32800345 10.1016/j.bbrc.2020.07.030

[141] Y. Gao , Y. Wang , L. Sun , Q. Meng , L. Cai , and X. Dong , “Expression of TGFβ‐1 and EHD1 Correlated With Survival of Non‐Small Cell Lung Cancer,” Tumour Biology 35, no. 9 (2014): 9371–9380. 24946721 10.1007/s13277-014-2164-x

[142] K. Y. Huang , H. C. Yu , M. C. Lu , et al., “Identification of a Novel Eps 15 Homology Domain‐Containing Protein 1 (EHD1) and EHD4‐Binding Motif in Phostensin,” Journal of Biochemistry 177, no. 4 (2025): 297–304. 39776131 10.1093/jb/mvaf002

[143] Z. Z. Wu , H. P. Lu , and C. C. Chao , “Identification and Functional Analysis of Genes Which Confer Resistance to Cisplatin in Tumor Cells,” Biochemical Pharmacology 80, no. 2 (2010): 262–276. 20361941 10.1016/j.bcp.2010.03.029

[144] J. Huang , X. Lan , T. Wang , et al., “Targeting the IL‐1β/EHD1/TUBB3 Axis Overcomes Resistance to EGFR‐TKI in NSCLC,” Oncogene 39, no. 8 (2020): 1739–1755. 31740781 10.1038/s41388-019-1099-5

[145] X. Wang , H. Yin , H. Zhang , et al., “NF‐kappaB‐Driven Improvement of EHD1 Contributes to Erlotinib Resistance in EGFR‐Mutant Lung Cancers,” Cell Death & Disease 9, no. 4 (2018): 418. 29549343 10.1038/s41419-018-0447-7 PMC5856828

[146] F. Tian , J. Huang , W. Fan , et al., “M(6)A‐Modified EHD1 Controls PD‐L1 Endosomal Trafficking to Modulate Immune Evasion and Immunotherapy Responses in Lung Adenocarcinoma,” Cancer Communications 45, no. 10 (2025): 1285–1308. 40703029 10.1002/cac2.70052 PMC12531423

[147] W. W. Shen , I. Bièche , L. Fuhrmann , et al., “EHD2 Is a Predictive Biomarker of Chemotherapy Efficacy in Triple Negative Breast Carcinoma,” Scientific Reports 10, no. 1 (2020): 7998. 32409676 10.1038/s41598-020-65054-5 PMC7224205

[148] Z. Zhang , J. Chen , X. Huo , et al., “Identification of a Mesenchymal‐Related Signature Associated With Clinical Prognosis in Glioma,” Aging 13, no. 9 (2021): 12431–12455. 33875619 10.18632/aging.202886 PMC8148476

[149] Y. Chen , S. Xue , J. Shi , C. He , and Q. Zhang , “EHD2, a Novel HIF Target Gene, Is a Promising Biomarker in Clear Cell Renal Cell Carcinoma,” International Journal of Clinical and Experimental Pathology 16, no. 11 (2023): 344–351. 38059172 PMC10695750

[150] B. Chen , Z. Jiao , X. Yin , Z. Qian , J. Gu , and H. Sun , “Novel Insights Into Biomarkers Associated With Renal Cell Carcinoma,” Oncology Letters 16, no. 1 (2018): 83–90. 29928389 10.3892/ol.2018.8665 PMC6006415

[151] Y. Kim , M. H. Kim , S. Jeon , et al., “Prognostic Implication of Histological Features Associated With EHD2 Expression in Papillary Thyroid Carcinoma,” PLoS One 12, no. 3 (2017): e0174737. 28358874 10.1371/journal.pone.0174737 PMC5373597

[152] K. Davalieva , I. M. Kostovska , S. Kiprijanovska , et al., “Proteomics Analysis of Malignant and Benign Prostate Tissue by 2D DIGE/MS Reveals New Insights Into Proteins Involved in Prostate Cancer,” Prostate 75, no. 14 (2015): 1586–1600. 26074449 10.1002/pros.23034

[153] S. Wei , J. Shao , J. Wang , et al., “EHD2 Inhibits the Invasive Ability of Lung Adenocarcinoma and Improves the Prognosis of Patients,” Journal of Thoracic Disease 14, no. 7 (2022): 2652–2664. 35928621 10.21037/jtd-22-842 PMC9344424

[154] L. Liu , M. Zhou , Z. Chen , and J. He , “Unsaturated Fatty Acid of Actinidia Chinesis Planch Seed Oil (Kiwi Fruit Essence) Inhibits Growth and Metastasis of Transplanted Tumor in Lung Adenocarcinoma Mice by Up‐Regulating EHD2 Expression,” Xi bao yu fen zi mian yi xue za zhi = Chinese Journal of Cellular and Molecular Immunology 36, no. 7 (2020): 622–627. 32727647

[155] F. Zhang , T. Zhang , Z. Zhao , Y. Ji , Y. Peng , and L. Zhao , “Circular RNA Eps15‐Homology Domain Containing Protein 2 Motivates Proliferation, Glycolysis but Refrains Autophagy in Non‐Small Cell Lung Cancer via Crosstalk With MicroRNA‐3186‐3p and Forkhead Box K1,” Bioengineered 13, no. 3 (2022): 6464–6475. 35220908 10.1080/21655979.2022.2031385 PMC8973962

[156] M. S. Zhang , J. D. Cui , D. Lee , et al., “Hypoxia‐Induced Macropinocytosis Represents a Metabolic Route for Liver Cancer,” Nature Communications 13, no. 1 (2022): 954. 10.1038/s41467-022-28618-9 PMC8854584 35177645

[157] H. Fan , T. Liu , H. Tian , and S. Zhang , “TUSC8 Inhibits the Development of Osteosarcoma by Sponging MiR‐197‐3p and Targeting EHD2,” International Journal of Molecular Medicine 46, no. 4 (2020): 1311–1320. 32945345 10.3892/ijmm.2020.4684 PMC7447318

[158] H. J. You , H. Y. Park , J. Kim , et al., “Integrative Radiogenomic Analysis for Genomic Signatures in Glioblastomas Presenting Leptomeningeal Dissemination,” Medicine 95, no. 27 (2016): e4109. 27399113 10.1097/MD.0000000000004109 PMC5058842

[159] C. Welinder , K. Pawłowski , A. M. Szasz , et al., “Correlation of Histopathologic Characteristics to Protein Expression and Function in Malignant Melanoma,” PLoS One 12, no. 4 (2017): e0176167. 28445515 10.1371/journal.pone.0176167 PMC5405986

[160] A. Yadav , B. Kumar , J. C. Lang , T. N. Teknos , and P. Kumar , “A Muscle‐Specific Protein ‘Myoferlin’ Modulates IL‐6/STAT3 Signaling by Chaperoning Activated STAT3 to Nucleus,” Oncogene 36, no. 46 (2017): 6374–6382. 28745314 10.1038/onc.2017.245 PMC5690845

[161] J. Li , J. Yang , L. Hua , et al., “Ese‐3 Contributes to Colon Cancer Progression by Downregulating EHD2 and Transactivating INPP4B,” American Journal of Cancer Research 11, no. 1 (2021): 92–107. 33520362 PMC7840712

[162] C. Guan , C. Lu , M. Xiao , and W. Chen , “EHD2 Overexpression Suppresses the Proliferation, Migration, and Invasion in Human Colon Cancer,” Cancer Investigation 39, no. 4 (2021): 297–309. 33356637 10.1080/07357907.2020.1870125

[163] M. Nakakido , K. Tamura , S. Chung , et al., “Phosphatidylinositol Glycan Anchor Biosynthesis, Class X Containing Complex Promotes Cancer Cell Proliferation Through Suppression of EHD2 and ZIC1, Putative Tumor Suppressors,” International Journal of Oncology 49, no. 3 (2016): 868–876. 27572108 10.3892/ijo.2016.3607 PMC4948962

[164] M. Ye , G. Fan , S. Zhu , W. Han , and Y. Xie , “Low Expressions of EHD2 and E‐Cadherin Correlate With a Poor Prognosis for Clear Cell Renal Cell Carcinoma,” Zhong Nan Da Xue Xue Bao. Yi Xue Ban = Journal of Central South University. Medical Sciences 44, no. 8 (2019): 864–870. 31570672 10.11817/j.issn.1672-7347.2019.190098

[165] Y. Shao , M. Yu , L. Zhang , et al., “In‐Depth Analysis of Lymph Node Metastasis‐Related Sialylated Protein Profiling and Their Clinical and Biological Significance in Colorectal Cancer Using Mass Spectrometry and Multi‐Omics Technologies,” Scientific Reports 14, no. 1 (2024): 28535. 39558044 10.1038/s41598-024-79893-z PMC11574123

[166] S. Li , W. Qi , J. Wu , et al., “A Comprehensive Study Employing Computational Analysis and Mendelian Randomization Has Revealed the Impact of Key Genes on Liver Cancer,” Biomedicine 13, no. 6 (2025): 1313. 10.3390/biomedicines13061313 PMC12190003 40564032

[167] S. Xie , C. Dierlam , E. Smith , et al., “The Retromer Complex Regulates *C. elegans* Development and Mammalian Ciliogenesis,” Journal of Cell Science 135, no. 10 (2022): jcs259396. 35510502 10.1242/jcs.259396 PMC9189432

[168] S. Xie , N. Naslavsky , and S. Caplan , “Emerging Insights Into CP110 Removal During Early Steps of Ciliogenesis,” Journal of Cell Science 137, no. 4 (2024), 10.1242/jcs.261579. PMC10941660 38415788

[169] M. Imamura , A. Takahashi , M. Matsunami , et al., “Genome‐Wide Association Studies Identify Two Novel Loci Conferring Susceptibility to Diabetic Retinopathy in Japanese Patients With Type 2 Diabetes,” Human Molecular Genetics 30, no. 8 (2021): 716–726. 33607655 10.1093/hmg/ddab044 PMC9022108

[170] M. A. Rainey , M. George , G. Ying , et al., “The Endocytic Recycling Regulator EHD1 Is Essential for Spermatogenesis and Male Fertility in Mice,” BMC Developmental Biology 10 (2010): 37. 20359371 10.1186/1471-213X-10-37 PMC2856533

[171] M. George , M. A. Rainey , M. Naramura , et al., “Ehd4 Is Required to Attain Normal Prepubertal Testis Size but Dispensable for Fertility in Male Mice,” Genesis 48, no. 5 (2010): 328–342. 20213691 10.1002/dvg.20620 PMC2913599

[172] L. Zhang , L. Ding , Y. Li , et al., “EHD3 Positively Regulated by NR5A1 Participates in Testosterone Synthesis via Endocytosis,” Life Sciences 278 (2021): 119570. 33964295 10.1016/j.lfs.2021.119570

[173] J. Patrakka , Z. Xiao , M. Nukui , et al., “Expression and Subcellular Distribution of Novel Glomerulus‐Associated Proteins Dendrin, Ehd3, Sh2d4a, Plekhh2, and 2310066E14Rik,” Journal of the American Society of Nephrology 18, no. 3 (2007): 689–697. 17251388 10.1681/ASN.2006060675

[174] N. Karaiskos , M. Rahmatollahi , A. Boltengagen , et al., “A Single‐Cell Transcriptome Atlas of the Mouse Glomerulus,” Journal of the American Society of Nephrology 29, no. 8 (2018): 2060–2068. 29794128 10.1681/ASN.2018030238 PMC6065081

[175] N. C. Finch , S. S. Fawaz , C. R. Neal , et al., “Reduced Glomerular Filtration in Diabetes Is Attributable to Loss of Density and Increased Resistance of Glomerular Endothelial Cell Fenestrations,” Journal of the American Society of Nephrology 33, no. 6 (2022): 1120–1136. 35292439 10.1681/ASN.2021030294 PMC9161794

[176] M. George , M. A. Rainey , M. Naramura , et al., “Renal Thrombotic Microangiopathy in Mice With Combined Deletion of Endocytic Recycling Regulators EHD3 and EHD4,” PLoS One 6, no. 3 (2011): e17838. 21408024 10.1371/journal.pone.0017838 PMC3052385

[177] C. Fryklund , B. Morén , S. Shah , et al., “EH Domain‐Containing 2 Deficiency Restricts Adipose Tissue Expansion and Impairs Lipolysis in Primary Inguinal Adipocytes,” Frontiers in Physiology 12 (2021): 740666. 34630160 10.3389/fphys.2021.740666 PMC8497890

[178] M. Neuhaus , C. Fryklund , H. Taylor , et al., “EHD2 Regulates Plasma Membrane Integrity and Downstream Insulin Receptor Signaling Events,” Molecular Biology of the Cell 34, no. 12 (2023): ar124. 37703099 10.1091/mbc.E23-03-0078 PMC10846623

[179] L. Sackmann‐Sala , D. E. Berryman , E. R. Lubbers , et al., “Age‐Related and Depot‐Specific Changes in White Adipose Tissue of Growth Hormone Receptor‐Null Mice,” Journals of Gerontology. Series A, Biological Sciences and Medical Sciences 69, no. 1 (2014): 34–43. 23873966 10.1093/gerona/glt110 PMC3859361

[180] G. Fan , M. Kaßmann , Y. Cui , et al., “Age Attenuates the T‐Type Ca(V) 3.2‐RyR Axis in Vascular Smooth Muscle,” Aging Cell 19, no. 4 (2020): e13134. 32187825 10.1111/acel.13134 PMC7189999

[181] V. Buggia‐Prevot , C. G. Fernandez , V. Udayar , et al., “A Function for EHD Family Proteins in Unidirectional Retrograde Dendritic Transport of BACE1 and Alzheimer's Disease Aβ Production,” Cell Reports 5, no. 6 (2013): 1552–1563. 24373286 10.1016/j.celrep.2013.12.006 PMC3932704

[182] Y. Olswang‐Kutz , Y. Gertel , S. Benjamin , et al., “ *Drosophila* Past1 Is Involved in Endocytosis and Is Required for Germline Development and Survival of the Adult Fly,” Journal of Cell Science 122, no. Pt 4 (2009): 471–480. 19174465 10.1242/jcs.038521

[183] C. A. Poodry , L. Hall , and D. T. Suzuki , “Developmental Properties of Shibire: A Pleiotropic Mutation Affecting Larval and Adult Locomotion and Development,” Developmental Biology 32, no. 2 (1973): 373–386. 4208027 10.1016/0012-1606(73)90248-0

[184] C. A. Smith , S. E. Dho , J. Donaldson , U. Tepass , and C. J. McGlade , “The Cell Fate Determinant Numb Interacts With EHD/Rme‐1 Family Proteins and Has a Role in Endocytic Recycling,” Molecular Biology of the Cell 15, no. 8 (2004): 3698–3708. 15155807 10.1091/mbc.E04-01-0026 PMC491829

[185] O. Dorot , H. Steller , D. Segal , and M. Horowitz , “Past1 Modulates *Drosophila* Eye Development,” PLoS One 12, no. 1 (2017): e0169639. 28060904 10.1371/journal.pone.0169639 PMC5218476

[186] C. J. Ma , Y. Yang , T. Kim , et al., “An Early Endosome‐Derived Retrograde Trafficking Pathway Promotes Secretory Granule Maturation,” Journal of Cell Biology 219, no. 3 (2020): e201808017. 32045479 10.1083/jcb.201808017 PMC7055004

[187] B. Grant and D. Hirsh , “Receptor‐Mediated Endocytosis in the *Caenorhabditis elegans* Oocyte,” Molecular Biology of the Cell 10, no. 12 (1999): 4311–4326. 10588660 10.1091/mbc.10.12.4311 PMC25760

[188] C. Zhai and M. Q. Dong , “RME‐1‐Associated Recycling Endosomes Participate in Vitellogenin Secretion in *Caenorhabditis elegans* ,” Life Metabolism 4, no. 6 (2025): loaf026. 41070192 10.1093/lifemeta/loaf026 PMC12507027

[189] A. Shi , S. Pant , Z. Balklava , C. C. Chen , V. Figueroa , and B. D. Grant , “A Novel Requirement for *C. elegans* Alix/ALX‐1 in RME‐1‐Mediated Membrane Transport,” Current Biology 17, no. 22 (2007): 1913–1924. 17997305 10.1016/j.cub.2007.10.045 PMC2175126

[190] C. C. Chen , P. J. Schweinsberg , S. Vashist , D. P. Mareiniss , E. J. Lambie , and B. D. Grant , “RAB‐10 Is Required for Endocytic Recycling in the *Caenorhabditis elegans* Intestine,” Molecular Biology of the Cell 17, no. 3 (2006): 1286–1297. 16394106 10.1091/mbc.E05-08-0787 PMC1382317

[191] L. Nilsson , B. Conradt , A. F. Ruaud , et al., “ *Caenorhabditis elegans* Num‐1 Negatively Regulates Endocytic Recycling,” Genetics 179, no. 1 (2008): 375–387. 18493060 10.1534/genetics.108.087247 PMC2390616

[192] S. Pant , M. Sharma , K. Patel , S. Caplan , C. M. Carr , and B. D. Grant , “AMPH‐1/Amphiphysin/Bin1 Functions With RME‐1/Ehd1 in Endocytic Recycling,” Nature Cell Biology 11, no. 12 (2009): 1399–1410. 19915558 10.1038/ncb1986 PMC2788952

[193] X. Shi , F. Duan , L. Lin , Q. Xu , T. Xu , and R. Zhang , “WIP‐1 and DBN‐1 Promote Scission of Endocytic Vesicles by Bridging Actin and Dynamin‐1 in the *C. elegans* Intestine,” Journal of Cell Science 132, no. 12 (2019), 10.1242/jcs.228023. 31118234

[194] M. Bar , M. Sharfman , S. Schuster , and A. Avni , “The Coiled‐Coil Domain of EHD2 Mediates Inhibition of LeEix2 Endocytosis and Signaling,” PLoS One 4, no. 11 (2009): e7973. 19936242 10.1371/journal.pone.0007973 PMC2775675

[195] S. Mayor , J. F. Presley , and F. R. Maxfield , “Sorting of Membrane Components From Endosomes and Subsequent Recycling to the Cell Surface Occurs by a Bulk Flow Process,” Journal of Cell Biology 121, no. 6 (1993): 1257–1269. 8509447 10.1083/jcb.121.6.1257 PMC2119709

[196] I. Mellman , “Endocytosis and Molecular Sorting,” Annual Review of Cell and Developmental Biology 12 (1996): 575–625. 10.1146/annurev.cellbio.12.1.575 8970738

[197] M. Jovic , M. Sharma , J. Rahajeng , and S. Caplan , “The Early Endosome: A Busy Sorting Station for Proteins at the Crossroads,” Histology and Histopathology 25, no. 1 (2010): 99–112. 19924646 10.14670/hh-25.99 PMC2810677

[198] R. Yarwood , J. Hellicar , P. G. Woodman , and M. Lowe , “Membrane Trafficking in Health and Disease,” Disease Models & Mechanisms 13, no. 4 (2020): dmm043448. 32433026 10.1242/dmm.043448 PMC7197876

[199] S. D. Conner and S. L. Schmid , “Regulated Portals of Entry Into the Cell,” Nature 422, no. 6927 (2003): 37–44. 12621426 10.1038/nature01451

[200] P. D. Allaire , A. L. Marat , C. Dall'Armi , G. Di Paolo , P. S. McPherson , and B. Ritter , “The Connecdenn DENN Domain: A GEF for Rab35 Mediating Cargo‐Specific Exit From Early Endosomes,” Molecular Cell 37, no. 3 (2010): 370–382. 20159556 10.1016/j.molcel.2009.12.037 PMC2825348

[201] J. Rahajeng , S. S. Giridharan , N. Naslavsky , and S. Caplan , “Collapsin Response Mediator Protein‐2 (Crmp2) Regulates Trafficking by Linking Endocytic Regulatory Proteins to Dynein Motors,” Journal of Biological Chemistry 285, no. 42 (2010): 31918–31922. 20801876 10.1074/jbc.C110.166066 PMC2952192

[202] M. Sato , K. Sato , W. Liou , S. Pant , A. Harada , and B. D. Grant , “Regulation of Endocytic Recycling by *C. elegans* Rab35 and Its Regulator RME‐4, a Coated‐Pit Protein,” EMBO Journal 27, no. 8 (2008): 1183–1196. 18354496 10.1038/emboj.2008.54 PMC2367398

[203] T. Jones , N. Naslavsky , and S. Caplan , “Eps15 Homology Domain Protein 4 (EHD4) is Required for Eps15 Homology Domain Protein 1 (EHD1)‐Mediated Endosomal Recruitment and Fission,” PLoS One 15, no. 9 (2020): e0239657. 32966336 10.1371/journal.pone.0239657 PMC7511005

[204] B. Cai , S. S. Giridharan , J. Zhang , et al., “Differential Roles of C‐Terminal Eps15 Homology Domain Proteins as Vesiculators and Tubulators of Recycling Endosomes,” Journal of Biological Chemistry 288, no. 42 (2013): 30172–30180. 24019528 10.1074/jbc.M113.488627 PMC3798485

[205] E. Galperin , S. Benjamin , D. Rapaport , R. Rotem‐Yehudar , S. Tolchinsky , and M. Horowitz , “EHD3: A Protein That Resides in Recycling Tubular and Vesicular Membrane Structures and Interacts With EHD1,” Traffic 3, no. 8 (2002): 575–589. 12121420 10.1034/j.1600-0854.2002.30807.x

[206] M. George , G. Ying , M. A. Rainey , et al., “Shared as Well as Distinct Roles of EHD Proteins Revealed by Biochemical and Functional Comparisons in Mammalian Cells and *C. elegans* ,” BMC Cell Biology 8 (2007): 3. 17233914 10.1186/1471-2121-8-3 PMC1793994

[207] J. E. McKenzie , B. Raisley , X. Zhou , et al., “Retromer Guides STxB and CD8‐M6PR From Early to Recycling Endosomes, EHD1 Guides STxB From Recycling Endosome to Golgi,” Traffic 13, no. 8 (2012): 1140–1159. 22540229 10.1111/j.1600-0854.2012.01374.x PMC3396774

[208] J. Zhang , C. Reiling , J. B. Reinecke , et al., “Rabankyrin‐5 Interacts With EHD1 and Vps26 to Regulate Endocytic Trafficking and Retromer Function,” Traffic 13, no. 5 (2012): 745–757. 22284051 10.1111/j.1600-0854.2012.01334.x PMC3613124

[209] J. Zhang , N. Naslavsky , and S. Caplan , “EHDs Meet the Retromer: Complex Regulation of Retrograde Transport,” Cellular Logistics 2, no. 3 (2012): 161–165. 23181199 10.4161/cl.20582 PMC3498075

[210] K. Bahl , S. Xie , G. Spagnol , P. Sorgen , N. Naslavsky , and S. Caplan , “EHD3 Protein Is Required for Tubular Recycling Endosome Stabilization, and an Asparagine‐Glutamic Acid Residue Pair Within Its Eps15 Homology (EH) Domain Dictates Its Selective Binding to NPF Peptides,” Journal of Biological Chemistry 291, no. 26 (2016): 13465–13478. 27189942 10.1074/jbc.M116.716407 PMC4919434

[211] S. Mayor , R. G. Parton , and J. G. Donaldson , “Clathrin‐Independent Pathways of Endocytosis,” Cold Spring Harbor Perspectives in Biology 6, no. 6 (2014): a016758. 24890511 10.1101/cshperspect.a016758 PMC4031960

[212] L. Pelkmans , E. Fava , H. Grabner , et al., “Genome‐Wide Analysis of Human Kinases in Clathrin‐ and Caveolae/Raft‐Mediated Endocytosis,” Nature 436, no. 7047 (2005): 78–86. 15889048 10.1038/nature03571

[213] E. Boucrot , M. T. Howes , T. Kirchhausen , and R. G. Parton , “Redistribution of Caveolae During Mitosis,” Journal of Cell Science 124, no. Pt 12 (2011): 1965–1972. 21625007 10.1242/jcs.076570 PMC3104031

[214] L. Pelkmans and A. Helenius , “Endocytosis via Caveolae,” Traffic 3, no. 5 (2002): 311–320. 11967125 10.1034/j.1600-0854.2002.30501.x

[215] P. F. Pilch , T. Meshulam , S. Ding , and L. Liu , “Caveolae and Lipid Trafficking in Adipocytes,” Clinical Lipidology 6, no. 1 (2011): 49–58. 21625349 10.2217/clp.10.80 PMC3103140

[216] B. Sinha , D. Koster , R. Ruez , et al., “Cells Respond to Mechanical Stress by Rapid Disassembly of Caveolae,” Cell 144, no. 3 (2011): 402–413. 21295700 10.1016/j.cell.2010.12.031 PMC3042189

[217] J. Lee and G. W. Schmid‐Schonbein , “Biomechanics of Skeletal Muscle Capillaries: Hemodynamic Resistance, Endothelial Distensibility, and Pseudopod Formation,” Annals of Biomedical Engineering 23, no. 3 (1995): 226–246. 7631979 10.1007/BF02584425

[218] O. L. Gervasio , W. D. Phillips , L. Cole , and D. G. Allen , “Caveolae Respond to Cell Stretch and Contribute to Stretch‐Induced Signaling,” Journal of Cell Science 124, no. Pt 21 (2011): 3581–3590. 22045729 10.1242/jcs.084376

[219] P. Sens and M. S. Turner , “Budded Membrane Microdomains as Tension Regulators,” Physical Review. E, Statistical, Nonlinear, and Soft Matter Physics 73, no. 3 Pt 1 (2006): 031918. 16605569 10.1103/PhysRevE.73.031918

[220] T. Harder and K. Simons , “Caveolae, DIGs, and the Dynamics of Sphingolipid‐Cholesterol Microdomains,” Current Opinion in Cell Biology 9, no. 4 (1997): 534–542. 9261060 10.1016/s0955-0674(97)80030-0

[221] E. Ikonen and R. G. Parton , “Caveolins and Cellular Cholesterol Balance,” Traffic 1, no. 3 (2000): 212–217. 11208104 10.1034/j.1600-0854.2000.010303.x

[222] P. G. Frank , M. W. Cheung , S. Pavlides , G. Llaverias , D. S. Park , and M. P. Lisanti , “Caveolin‐1 and Regulation of Cellular Cholesterol Homeostasis,” American Journal of Physiology. Heart and Circulatory Physiology 291, no. 2 (2006): H677–H686. 16603689 10.1152/ajpheart.01092.2005

[223] Y. Fu , A. Hoang , G. Escher , R. G. Parton , Z. Krozowski , and D. Sviridov , “Expression of Caveolin‐1 Enhances Cholesterol Efflux in Hepatic Cells,” Journal of Biological Chemistry 279, no. 14 (2004): 14140–14146. 14729661 10.1074/jbc.M311061200

[224] N. Ariotti , M. A. Fernandez‐Rojo , Y. Zhou , et al., “Caveolae Regulate the Nanoscale Organization of the Plasma Membrane to Remotely Control Ras Signaling,” Journal of Cell Biology 204, no. 5 (2014): 777–792. 24567358 10.1083/jcb.201307055 PMC3941050

[225] N. Chaudhary , G. A. Gomez , M. T. Howes , et al., “Endocytic Crosstalk: Cavins, Caveolins, and Caveolae Regulate Clathrin‐Independent Endocytosis,” PLoS Biology 12, no. 4 (2014): e1001832. 24714042 10.1371/journal.pbio.1001832 PMC3979662

[226] B. Razani , S. E. Woodman , and M. P. Lisanti , “Caveolae: From Cell Biology to Animal Physiology,” Pharmacological Reviews 54, no. 3 (2002): 431–467. 12223531 10.1124/pr.54.3.431

[227] J. Couet , S. Li , T. Okamoto , T. Ikezu , and M. P. Lisanti , “Identification of Peptide and Protein Ligands for the Caveolin‐Scaffolding Domain. Implications for the Interaction of Caveolin With Caveolae‐Associated Proteins,” Journal of Biological Chemistry 272, no. 10 (1997): 6525–6533. 9045678 10.1074/jbc.272.10.6525

[228] G. Garcia‐Cardena , P. Martasek , B. S. Masters , et al., “Dissecting the Interaction Between Nitric Oxide Synthase (NOS) and Caveolin. Functional Significance of the Nos Caveolin Binding Domain In Vivo,” Journal of Biological Chemistry 272, no. 41 (1997): 25437–25440. 9325253 10.1074/jbc.272.41.25437

[229] U. Ortegren , M. Karlsson , N. Blazic , et al., “Lipids and Glycosphingolipids in Caveolae and Surrounding Plasma Membrane of Primary Rat Adipocytes,” European Journal of Biochemistry 271, no. 10 (2004): 2028–2036. 15128312 10.1111/j.1432-1033.2004.04117.x

[230] R. G. Parton , M. M. Kozlov , and N. Ariotti , “Caveolae and Lipid Sorting: Shaping the Cellular Response to Stress,” Journal of Cell Biology 219, no. 4 (2020): e20190507. 10.1083/jcb.201905071 PMC7147102 32328645

[231] G. A. Graf , P. M. Connell , D. R. van der Westhuyzen , and E. J. Smart , “The Class B, Type I Scavenger Receptor Promotes the Selective Uptake of High Density Lipoprotein Cholesterol Ethers Into Caveolae,” Journal of Biological Chemistry 274, no. 17 (1999): 12043–12048. 10207027 10.1074/jbc.274.17.12043

[232] I. M. Khater , F. Meng , T. H. Wong , I. R. Nabi , and G. Hamarneh , “Super Resolution Network Analysis Defines the Molecular Architecture of Caveolae and Caveolin‐1 Scaffolds,” Scientific Reports 8, no. 1 (2018): 9009. 29899348 10.1038/s41598-018-27216-4 PMC5998020

[233] M. Hubert , E. Larsson , and R. Lundmark , “Keeping in Touch With the Membrane; Protein‐ and Lipid‐Mediated Confinement of Caveolae to the Cell Surface,” Biochemical Society Transactions 48, no. 1 (2020): 155–163. 32049332 10.1042/BST20190386 PMC7054752

[234] R. G. Parton , “Caveolae: Structure, Function, and Relationship to Disease,” Annual Review of Cell and Developmental Biology 34 (2018): 111–136. 10.1146/annurev-cellbio-100617-062737 30296391

[235] P. J. Walser , N. Ariotti , M. Howes , et al., “Constitutive Formation of Caveolae in a Bacterium,” Cell 150, no. 4 (2012): 752–763. 22901807 10.1016/j.cell.2012.06.042

[236] Y. Gambin , N. Ariotti , K. A. McMahon , et al., “Single‐Molecule Analysis Reveals Self Assembly and Nanoscale Segregation of Two Distinct Cavin Subcomplexes on Caveolae,” eLife 3 (2013): e01434. 24473072 10.7554/eLife.01434 PMC3903133

[237] O. Kovtun , V. A. Tillu , N. Ariotti , R. G. Parton , and B. M. Collins , “Cavin Family Proteins and the Assembly of Caveolae,” Journal of Cell Science 128, no. 7 (2015): 1269–1278. 25829513 10.1242/jcs.167866 PMC4379724

[238] O. Kovtun , V. A. Tillu , W. Jung , et al., “Structural Insights Into the Organization of the Cavin Membrane Coat Complex,” Developmental Cell 31, no. 4 (2014): 405–419. 25453557 10.1016/j.devcel.2014.10.002

[239] B. Moren , C. Shah , M. T. Howes , et al., “EHD2 Regulates Caveolar Dynamics via ATP‐Driven Targeting and Oligomerization,” Molecular Biology of the Cell 23, no. 7 (2012): 1316–1329. 22323287 10.1091/mbc.E11-09-0787 PMC3315815

[240] C. G. Hansen , G. Howard , and B. J. Nichols , “Pacsin 2 Is Recruited to Caveolae and Functions in Caveolar Biogenesis,” Journal of Cell Science 124, no. Pt 16 (2011): 2777–2785. 21807942 10.1242/jcs.084319

[241] M. Stoeber , I. K. Stoeck , C. Hanni , C. K. Bleck , G. Balistreri , and A. Helenius , “Oligomers of the ATPase EHD2 Confine Caveolae to the Plasma Membrane Through Association With Actin,” EMBO Journal 31, no. 10 (2012): 2350–2364. 22505029 10.1038/emboj.2012.98 PMC3364743

[242] T. Nishimura and S. Shiro , “Super‐Resolution Analysis of PACSIN2 and EHD2 at Caveolae,” PLoS One 17, no. 7 (2022): e0271003. 35834519 10.1371/journal.pone.0271003 PMC9282494

[243] A. Ludwig , G. Howard , C. Mendoza‐Topaz , et al., “Molecular Composition and Ultrastructure of the Caveolar Coat Complex,” PLoS Biology 11, no. 8 (2013): e1001640. 24013648 10.1371/journal.pbio.1001640 PMC3754886

[244] A. Fujita , J. Cheng , K. Tauchi‐Sato , T. Takenawa , and T. Fujimoto , “A Distinct Pool of Phosphatidylinositol 4,5‐Bisphosphate in Caveolae Revealed by a Nanoscale Labeling Technique,” Proceedings of the National Academy of Sciences of the United States of America 106, no. 23 (2009): 9256–9261. 19470488 10.1073/pnas.0900216106 PMC2695096

[245] L. C. Simone , S. Caplan , and N. Naslavsky , “Role of Phosphatidylinositol 4,5‐Bisphosphate in Regulating EHD2 Plasma Membrane Localization,” PLoS One 8, no. 9 (2013): e74519. 24040268 10.1371/journal.pone.0074519 PMC3769341

[246] J. Mohan , B. Moren , E. Larsson , M. R. Holst , and R. Lundmark , “Cavin3 Interacts With cavin1 and caveolin1 to Increase Surface Dynamics of Caveolae,” Journal of Cell Science 128, no. 5 (2015): 979–991. 25588833 10.1242/jcs.161463

[247] Y. Senju , E. Rosenbaum , C. Shah , et al., “Phosphorylation of PACSIN2 by Protein Kinase C Triggers the Removal of Caveolae From the Plasma Membrane,” Journal of Cell Science 128, no. 15 (2015): 2766–2780. 26092940 10.1242/jcs.167775

[248] C. Matthaeus , K. A. Sochacki , A. M. Dickey , et al., “The Molecular Organization of Differentially Curved Caveolae Indicates Bendable Structural Units at the Plasma Membrane,” Nature Communications 13, no. 1 (2022): 7234. 10.1038/s41467-022-34958-3 PMC9700719 36433988

[249] M. Hubert , E. Larsson , N. V. G. Vegesna , et al., “Lipid Accumulation Controls the Balance Between Surface Connection and Scission of Caveolae,” eLife 9 (2020): e55038. 32364496 10.7554/eLife.55038 PMC7239661

[250] M. Lenz , S. Morlot , and A. Roux , “Mechanical Requirements for Membrane Fission: Common Facts From Various Examples,” FEBS Letters 583, no. 23 (2009): 3839–3846. 19903475 10.1016/j.febslet.2009.11.012

[251] I. Yeow , G. Howard , J. Chadwick , et al., “EHD Proteins Cooperate to Generate Caveolar Clusters and to Maintain Caveolae During Repeated Mechanical Stress,” Current Biology 27, no. 19 (2017): 2951–2962.e5. 28943089 10.1016/j.cub.2017.07.047 PMC5640515

[252] O. Daumke , R. Lundmark , Y. Vallis , S. Martens , P. J. Butler , and H. T. McMahon , “Architectural and Mechanistic Insights Into an EHD ATPase Involved in Membrane Remodelling,” Nature 449, no. 7164 (2007): 923–927. 17914359 10.1038/nature06173

[253] S. Le Lay , E. Hajduch , M. R. Lindsay , et al., “Cholesterol‐Induced Caveolin Targeting to Lipid Droplets in Adipocytes: A Role for Caveolar Endocytosis,” Traffic 7, no. 5 (2006): 549–561. 16643278 10.1111/j.1600-0854.2006.00406.x

[254] D. K. Sharma , A. Choudhury , R. D. Singh , C. L. Wheatley , D. L. Marks , and R. E. Pagano , “Glycosphingolipids Internalized via Caveolar‐Related Endocytosis Rapidly Merge With the Clathrin Pathway in Early Endosomes and Form Microdomains for Recycling,” Journal of Biological Chemistry 278, no. 9 (2003): 7564–7572. 12482757 10.1074/jbc.M210457200

[255] D. K. Sharma , J. C. Brown , Z. Cheng , E. L. Holicky , D. L. Marks , and R. E. Pagano , “The Glycosphingolipid, Lactosylceramide, Regulates Beta1‐Integrin Clustering and Endocytosis,” Cancer Research 65, no. 18 (2005): 8233–8241. 16166299 10.1158/0008-5472.CAN-05-0803

[256] D. K. Sharma , J. C. Brown , A. Choudhury , et al., “Selective Stimulation of Caveolar Endocytosis by Glycosphingolipids and Cholesterol,” Molecular Biology of the Cell 15, no. 7 (2004): 3114–3122. 15107466 10.1091/mbc.E04-03-0189 PMC452569

[257] S. Torrino , W. W. Shen , C. M. Blouin , et al., “EHD2 Is a Mechanotransducer Connecting Caveolae Dynamics With Gene Transcription,” Journal of Cell Biology 217, no. 12 (2018): 4092–4105. 30348749 10.1083/jcb.201801122 PMC6279385

[258] L. Wang and B. D. Dynlacht , “The Regulation of Cilium Assembly and Disassembly in Development and Disease,” Development 145, no. 18 (2018): dev151407. 30224385 10.1242/dev.151407 PMC6176931

[259] P. Mill , S. T. Christensen , and L. B. Pedersen , “Primary Cilia as Dynamic and Diverse Signalling Hubs in Development and Disease,” Nature Reviews. Genetics 24, no. 7 (2023): 421–441. 10.1038/s41576-023-00587-9 PMC7615029 37072495

[260] J. F. Reiter and M. R. Leroux , “Genes and Molecular Pathways Underpinning Ciliopathies,” Nature Reviews. Molecular Cell Biology 18, no. 9 (2017): 533–547. 28698599 10.1038/nrm.2017.60 PMC5851292

[261] M. V. Nachury , A. V. Loktev , Q. Zhang , et al., “A Core Complex of BBS Proteins Cooperates With the GTPase Rab8 to Promote Ciliary Membrane Biogenesis,” Cell 129, no. 6 (2007): 1201–1213. 17574030 10.1016/j.cell.2007.03.053

[262] C. J. Westlake , L. M. Baye , M. V. Nachury , et al., “Primary Cilia Membrane Assembly Is Initiated by Rab11 and Transport Protein Particle II (TRAPPII) Complex‐Dependent Trafficking of Rabin8 to the Centrosome,” Proceedings of the National Academy of Sciences of the United States of America 108, no. 7 (2011): 2759–2764. 21273506 10.1073/pnas.1018823108 PMC3041065

[263] S. Yoshimura , J. Egerer , E. Fuchs , A. K. Haas , and F. A. Barr , “Functional Dissection of Rab GTPases Involved in Primary Cilium Formation,” Journal of Cell Biology 178, no. 3 (2007): 363–369. 17646400 10.1083/jcb.200703047 PMC2064854

[264] A. Knodler , S. Feng , J. Zhang , et al., “Coordination of Rab8 and Rab11 in Primary Ciliogenesis,” Proceedings of the National Academy of Sciences of the United States of America 107, no. 14 (2010): 6346–6351. 20308558 10.1073/pnas.1002401107 PMC2851980

[265] C. T. Wu , H. Y. Chen , and T. K. Tang , “Myosin‐Va Is Required for Preciliary Vesicle Transportation to the Mother Centriole During Ciliogenesis,” Nature Cell Biology 20, no. 2 (2018): 175–185. 29335527 10.1038/s41556-017-0018-7

[266] A. Spektor , W. Y. Tsang , D. Khoo , and B. D. Dynlacht , “Cep97 and CP110 Suppress a Cilia Assembly Program,” Cell 130, no. 4 (2007): 678–690. 17719545 10.1016/j.cell.2007.06.027

[267] S. Sorokin , “Centrioles and the Formation of Rudimentary Cilia by Fibroblasts and Smooth Muscle Cells,” Journal of Cell Biology 15 (1962): 363–377. 13978319 10.1083/jcb.15.2.363 PMC2106144

[268] S. P. Sorokin , “Reconstructions of Centriole Formation and Ciliogenesis in Mammalian Lungs,” Journal of Cell Science 3, no. 2 (1968): 207–230. 5661997 10.1242/jcs.3.2.207

[269] S. Xie , N. Naslavsky , and S. Caplan , “EHD1 Promotes CP110 Ubiquitination by Centriolar Satellite Delivery of HERC2 to the Mother Centriole,” EMBO Reports 4, no. 6 (2023): e56317. 10.15252/embr.202256317 PMC10240189 37074924

[270] V. Quarantotti , J. X. Chen , J. Tischer , et al., “Centriolar Satellites Are Acentriolar Assemblies of Centrosomal Proteins,” EMBO Journal 38, no. 14 (2019): e101082. 31304626 10.15252/embj.2018101082 PMC6627235

[271] B. Ashraf , J. Reddick‐Umoja , J. Grant , J. Iyer , N. Naslavsky , and S. Caplan , “The Endocytic Fission Protein EHD1 Interacts With Tubulin and Regulates Microtubule Function,” Biochimica et Biophysica Acta, Molecular Cell Research 1873, no. 3 (2026): 120120. 41611020 10.1016/j.bbamcr.2026.120120 PMC13150490

[272] C. Insinna , Q. Lu , I. Teixeira , et al., “Investigation of F‐BAR Domain PACSIN Proteins Uncovers Membrane Tubulation Function in Cilia Assembly and Transport,” Nature Communications 10, no. 1 (2019): 428. 10.1038/s41467-018-08192-9 PMC6347608 30683896

[273] E. A. Nigg , “Cell Biology: A Licence for Duplication,” Nature 442, no. 7105 (2006): 874–875. 16929283 10.1038/442874a

[274] M. F. Tsou and T. Stearns , “Mechanism Limiting Centrosome Duplication to Once Per Cell Cycle,” Nature 442, no. 7105 (2006): 947–951. 16862117 10.1038/nature04985

[275] B. R. Mardin and E. Schiebel , “Breaking the Ties That Bind: New Advances in Centrosome Biology,” Journal of Cell Biology 197, no. 1 (2012): 11–18. 22472437 10.1083/jcb.201108006 PMC3317805

[276] K. Lee and K. Rhee , “Separase‐Dependent Cleavage of Pericentrin B Is Necessary and Sufficient for Centriole Disengagement During Mitosis,” Cell Cycle 11, no. 13 (2012): 2476–2485. 22722493 10.4161/cc.20878

[277] J. K. Pagan , A. Marzio , M. J. Jones , et al., “Degradation of Cep68 and PCNT Cleavage Mediate Cep215 Removal From the PCM to Allow Centriole Separation, Disengagement and Licensing,” Nature Cell Biology 17, no. 1 (2015): 31–43. 25503564 10.1038/ncb3076 PMC4415623

[278] N. Naslavsky and S. Caplan , “Endocytic Membrane Trafficking in the Control of Centrosome Function,” Current Opinion in Cell Biology 65 (2020): 150–155. 32143977 10.1016/j.ceb.2020.01.009 PMC7483210

[279] H. Hehnly , C. T. Chen , C. M. Powers , H. L. Liu , and S. Doxsey , “The Centrosome Regulates the Rab11‐ Dependent Recycling Endosome Pathway at Appendages of the Mother Centriole,” Current Biology 22, no. 20 (2012): 1944–1950. 22981775 10.1016/j.cub.2012.08.022 PMC3917512

[280] H. Hehnly and S. Doxsey , “Rab11 Endosomes Contribute to Mitotic Spindle Organization and Orientation,” Developmental Cell 28, no. 5 (2014): 497–507. 24561039 10.1016/j.devcel.2014.01.014 PMC4030695

[281] A. Gromley , C. Yeaman , J. Rosa , et al., “Centriolin Anchoring of Exocyst and SNARE Complexes at the Midbody Is Required for Secretory‐Vesicle‐Mediated Abscission,” Cell 123, no. 1 (2005): 75–87. 16213214 10.1016/j.cell.2005.07.027

[282] S. Xie , J. B. Reinecke , T. Farmer , et al., “Vesicular Trafficking Plays a Role in Centriole Disengagement and Duplication,” Molecular Biology of the Cell 29, no. 22 (2018): 2622–2631. 30188792 10.1091/mbc.E18-04-0241 PMC6249839

[283] J. B. Reinecke , D. Katafiasz , N. Naslavsky , and S. Caplan , “Novel Functions for the Endocytic Regulatory Proteins MICAL‐L1 and EHD1 in Mitosis,” Traffic 16, no. 1 (2015): 48–67. 25287187 10.1111/tra.12234 PMC4275409

[284] D. C. Chan , “Fusion and Fission: Interlinked Processes Critical for Mitochondrial Health,” Annual Review of Genetics 46 (2012): 265–287. 10.1146/annurev-genet-110410-132529 22934639

[285] K. B. Heine and W. R. Hood , “Mitochondrial Behaviour, Morphology, and Animal Performance,” Biological Reviews of the Cambridge Philosophical Society 95, no. 3 (2020): 730–737. 32022456 10.1111/brv.12584

[286] X. Wang , B. Su , S. L. Siedlak , et al., “Amyloid‐Beta Overproduction Causes Abnormal Mitochondrial Dynamics via Differential Modulation of Mitochondrial Fission/Fusion Proteins,” Proceedings of the National Academy of Sciences of the United States of America 105, no. 49 (2008): 19318–19323. 19050078 10.1073/pnas.0804871105 PMC2614759

[287] M. H. Yan , X. Wang , and X. Zhu , “Mitochondrial Defects and Oxidative Stress in Alzheimer Disease and Parkinson Disease,” Free Radical Biology & Medicine 62 (2013): 90–101. 23200807 10.1016/j.freeradbiomed.2012.11.014 PMC3744189

[288] F. Burte , V. Carelli , P. F. Chinnery , and P. Yu‐Wai‐Man , “Disturbed Mitochondrial Dynamics and Neurodegenerative Disorders,” Nature Reviews. Neurology 11, no. 1 (2015): 11–24. 25486875 10.1038/nrneurol.2014.228

[289] W. Wang , X. Wang , H. Fujioka , et al., “Parkinson's Disease‐Associated Mutant VPS35 Causes Mitochondrial Dysfunction by Recycling DLP1 Complexes,” Nature Medicine 22, no. 1 (2016): 54–63. 10.1038/nm.3983 PMC4826611 26618722

[290] W. Wang , X. Ma , L. Zhou , J. Liu , and X. Zhu , “A Conserved Retromer Sorting Motif Is Essential for Mitochondrial DLP1 Recycling by VPS35 in Parkinson's Disease Model,” Human Molecular Genetics 26, no. 4 (2017): 781–789. 28040727 10.1093/hmg/ddw430 PMC5903416

[291] W. Bleazard , J. M. McCaffery , E. J. King , et al., “The Dynamin‐Related GTPase Dnm1 Regulates Mitochondrial Fission in Yeast,” Nature Cell Biology 1, no. 5 (1999): 298–304. 10559943 10.1038/13014 PMC3739991

[292] A. M. Labrousse , M. D. Zappaterra , D. A. Rube , and A. M. van der Bliek , “ *C. elegans* Dynamin‐Related Protein DRP‐1 Controls Severing of the Mitochondrial Outer Membrane,” Molecular Cell 4, no. 5 (1999): 815–826. 10619028 10.1016/s1097-2765(00)80391-3

[293] T. Farmer , J. B. Reinecke , S. Xie , K. Bahl , N. Naslavsky , and S. Caplan , “Control of Mitochondrial Homeostasis by Endocytic Regulatory Proteins,” Journal of Cell Science 130, no. 14 (2017): 2359–2370. 28596240 10.1242/jcs.204537 PMC5536922

[294] M. Artal‐Sanz , W. Y. Tsang , E. M. Willems , et al., “The Mitochondrial Prohibitin Complex Is Essential for Embryonic Viability and Germline Function in *Caenorhabditis elegans* ,” Journal of Biological Chemistry 278, no. 34 (2003): 32091–32099. 12794069 10.1074/jbc.M304877200

[295] T. Jiang , J. Wang , C. Li , G. Cao , and X. Wang , “Prohibitins: A Key Link Between Mitochondria and Nervous System Diseases,” Oxidative Medicine and Cellular Longevity 2022 (2022): 7494863. 35847581 10.1155/2022/7494863 PMC9286927

[296] C. M. Blouin , S. Le Lay , A. Eberl , et al., “Lipid Droplet Analysis in Caveolin‐Deficient Adipocytes: Alterations in Surface Phospholipid Composition and Maturation Defects,” Journal of Lipid Research 51, no. 5 (2010): 945–956. 19965594 10.1194/jlr.M001016 PMC2853462

[297] R. Singh , S. Kaushik , Y. Wang , et al., “Autophagy Regulates Lipid Metabolism,” Nature 458, no. 7242 (2009): 1131–1135. 19339967 10.1038/nature07976 PMC2676208

[298] W. T. Wong , C. Schumacher , A. E. Salcini , et al., “A Protein‐Binding Domain, EH, Identified in the Receptor Tyrosine Kinase Substrate Eps15 and Conserved in Evolution,” Proceedings of the National Academy of Sciences of the United States of America 92, no. 21 (1995): 9530–9534. 7568168 10.1073/pnas.92.21.9530 PMC40835

[299] F. Fazioli , L. Minichiello , B. Matoskova , W. T. Wong , and P. P. Di Fiore , “Eps15, a Novel Tyrosine Kinase Substrate, Exhibits Transforming Activity,” Molecular and Cellular Biology 13, no. 9 (1993): 5814–5828. 7689153 10.1128/mcb.13.9.5814-5828.1993 PMC360326

[300] S. Confalonieri and P. P. Di Fiore , “The Eps15 Homology (EH) Domain,” FEBS Letters 513, no. 1 (2002): 24–29. 11911876 10.1016/s0014-5793(01)03241-0

[301] S. Paoluzi , L. Castagnoli , I. Lauro , et al., “Recognition Specificity of Individual EH Domains of Mammals and Yeast,” EMBO Journal 17, no. 22 (1998): 6541–6550. 9822599 10.1093/emboj/17.22.6541 PMC1171001

[302] A. E. Salcini , S. Confalonieri , M. Doria , et al., “Binding Specificity and In Vivo Targets of the EH Domain, a Novel Protein‐Protein Interaction Module,” Genes & Development 11, no. 17 (1997): 2239–2249. 9303539 10.1101/gad.11.17.2239 PMC275390

[303] T. de Beer , R. E. Carter , K. E. Lobel‐Rice , A. Sorkin , and M. Overduin , “Structure and Asn‐Pro‐Phe Binding Pocket of the Eps15 Homology Domain,” Science 281, no. 5381 (1998): 1357–1360. 9721102 10.1126/science.281.5381.1357

[304] T. de Beer , A. N. Hoofnagle , J. L. Enmon , et al., “Molecular Mechanism of NPF Recognition by EH Domains,” Nature Structural Biology 7, no. 11 (2000): 1018–1022. 11062555 10.1038/80924

[305] N. B. Miliaras and B. Wendland , “EH Proteins: Multivalent Regulators of Endocytosis (and Other Pathways),” Cell Biochemistry and Biophysics 41, no. 2 (2004): 295–318. 15475615 10.1385/CBB:41:2:295

[306] E. Santolini , A. E. Salcini , B. K. Kay , M. Yamabhai , and P. P. Di Fiore , “The EH Network,” Experimental Cell Research 253, no. 1 (1999): 186–209. 10579923 10.1006/excr.1999.4694

[307] U. Pohl , J. S. Smith , I. Tachibana , et al., “EHD2, EHD3, and EHD4 Encode Novel Members of a Highly Conserved Family of EH Domain‐Containing Proteins,” Genomics 63, no. 2 (2000): 255–262. 10673336 10.1006/geno.1999.6087

[308] K. Bahl , N. Naslavsky , and S. Caplan , “Role of the EHD2 Unstructured Loop in Dimerization, Protein Binding and Subcellular Localization,” PLoS One 10, no. 4 (2015): e0123710. 25875965 10.1371/journal.pone.0123710 PMC4398442

[309] M. Sharma , S. S. Giridharan , J. Rahajeng , N. Naslavsky , and S. Caplan , “MICAL‐L1 Links EHD1 to Tubular Recycling Endosomes and Regulates Receptor Recycling,” Molecular Biology of the Cell 20, no. 24 (2009): 5181–5194. 19864458 10.1091/mbc.E09-06-0535 PMC2793294

[310] F. Kieken , M. Sharma , M. Jovic , et al., “Mechanism for the Selective Interaction of C‐Terminal Eps15 Homology Domain Proteins With Specific Asn‐Pro‐Phe‐Containing Partners,” Journal of Biological Chemistry 285, no. 12 (2010): 8687–8694. 20106972 10.1074/jbc.M109.045666 PMC2838291

[311] Y. Xu , H. Shi , S. Wei , S. H. Wong , and W. Hong , “Mutually Exclusive Interactions of EHD1 With GS32 and Syndapin II,” Molecular Membrane Biology 21, no. 4 (2004): 269–277. 15371016 10.1080/09687680410001716871

[312] A. Braun , R. Pinyol , R. Dahlhaus , et al., “EHD Proteins Associate With Syndapin I and II and Such Interactions Play a Crucial Role in Endosomal Recycling,” Molecular Biology of the Cell 16, no. 8 (2005): 3642–3658. 15930129 10.1091/mbc.E05-01-0076 PMC1182304

[313] S. S. Giridharan , B. Cai , N. Vitale , N. Naslavsky , and S. Caplan , “Cooperation of MICAL‐L1, Syndapin2, and Phosphatidic Acid in Tubular Recycling Endosome Biogenesis,” Molecular Biology of the Cell 24, no. 11 (2013): 1776–1790, S1–S15. 10.1091/mbc.E13-01-0026 PMC3667729 23596323

[314] G. Ko , S. Paradise , H. Chen , et al., “Selective High‐Level Expression of Epsin 3 in Gastric Parietal Cells, Where It Is Localized at Endocytic Sites of Apical Canaliculi,” Proceedings of the National Academy of Sciences of the United States of America 107, no. 50 (2010): 21511–21516. 21115825 10.1073/pnas.1016390107 PMC3003030

[315] S. Gokool , D. Tattersall , and M. N. J. Seaman , “EHD1 Interacts With Retromer to Stabilize SNX1 Tubules and Facilitate Endosome‐To‐Golgi Retrieval,” Traffic 8, no. 12 (2007): 1873–1886. 17868075 10.1111/j.1600-0854.2007.00652.x

[316] I. Hernandez‐Perez , J. Rubio , A. Baumann , et al., “Kazrin Promotes Dynein/Dynactin‐Dependent Traffic From Early to Recycling Endosomes,” eLife (2023): 12, 10.7554/eLife.83793. PMC10181827 37096882

[317] S. Benjamin , H. Weidberg , D. Rapaport , et al., “EHD2 Mediates Trafficking From the Plasma Membrane by Modulating Rac1 Activity,” Biochemical Journal 439, no. 3 (2011): 433–442. 21756249 10.1042/BJ20111010

[318] F. Kieken , M. Jovic , N. Naslavsky , S. Caplan , and P. L. Sorgen , “EH Domain of EHD1,” Journal of Biomolecular NMR 39, no. 4 (2007): 323–329. 17899392 10.1007/s10858-007-9196-0

[319] G. D. Henry , D. J. Corrigan , J. V. Dineen , and J. D. Baleja , “Charge Effects in the Selection of NPF Motifs by the EH Domain of EHD1,” Biochemistry 49, no. 16 (2010): 3381–3392. 20329706 10.1021/bi100065r PMC2857698

[320] F. Kieken , M. Jovic , M. Tonelli , N. Naslavsky , S. Caplan , and P. L. Sorgen , “Structural Insight Into the Interaction of Proteins Containing NPF, DPF, and GPF Motifs With the C‐Terminal EH‐Domain of EHD1,” Protein Science 18, no. 12 (2009): 2471–2479. 19798736 10.1002/pro.258 PMC2821266

[321] J. J. Blume , A. Halbach , D. Behrendt , M. Paulsson , and M. Plomann , “EHD Proteins Are Associated With Tubular and Vesicular Compartments and Interact With Specific Phospholipids,” Experimental Cell Research 313, no. 2 (2007): 219–231. 17097635 10.1016/j.yexcr.2006.10.006

[322] Y. Posor , W. Jang , and V. Haucke , “Phosphoinositides as Membrane Organizers,” Nature Reviews. Molecular Cell Biology 23, no. 12 (2022): 797–816. 35589852 10.1038/s41580-022-00490-x PMC9117997

[323] N. Naslavsky , J. Rahajeng , S. Chenavas , P. L. Sorgen , and S. Caplan , “EHD1 and Eps15 Interact With Phosphatidylinositols via Their Eps15 Homology Domains,” Journal of Biological Chemistry 282, no. 22 (2007): 16612–16622. 17412695 10.1074/jbc.M609493200

[324] H. J. Santos , Y. Hanadate , K. Imai , H. Watanabe , and T. Nozaki , “ *Entamoeba histolytica* EHD1 Is Involved in Mitosome‐Endosome Contact,” MBio 13, no. 2 (2022): e0384921. 35404118 10.1128/mbio.03849-21 PMC9040739

[325] S. Lee , Y. Uchida , J. Wang , et al., “Transport Through Recycling Endosomes Requires EHD1 Recruitment by a Phosphatidylserine Translocase,” EMBO Journal 34, no. 5 (2015): 669–688. 25595798 10.15252/embj.201489703 PMC4365035

[326] R. Deo , M. S. Kushwah , S. C. Kamerkar , et al., “ATP‐Dependent Membrane Remodeling Links EHD1 Functions to Endocytic Recycling,” Nature Communications 9, no. 1 (2018): 5187. 10.1038/s41467-018-07586-z PMC6281616 30518883

[327] F. Campelo , G. Fabrikant , H. T. McMahon , and M. M. Kozlov , “Modeling Membrane Shaping by Proteins: Focus on EHD2 and N‐BAR Domains,” FEBS Letters 584, no. 9 (2010): 1830–1839. 19836393 10.1016/j.febslet.2009.10.023

[328] B. Cai , S. Xie , S. Caplan , and N. Naslavsky , “GRAF1 Forms a Complex With MICAL‐L1 and EHD1 to Cooperate in Tubular Recycling Endosome Vesiculation,” Frontiers in Cell and Development Biology 2 (2014): 22. 10.3389/fcell.2014.00022 PMC4214196 25364729

[329] S. C. Kamerkar , K. Roy , S. Bhattacharyya , and T. J. Pucadyil , “A Screen for Membrane Fission Catalysts Identifies the ATPase EHD1,” Biochemistry 58, no. 1 (2019): 65–71. 30403133 10.1021/acs.biochem.8b00925 PMC6327249

[330] V. A. Frolov , A. Escalada , S. A. Akimov , and A. V. Shnyrova , “Geometry of Membrane Fission,” Chemistry and Physics of Lipids 185 (2015): 129–140. 25062896 10.1016/j.chemphyslip.2014.07.006

[331] Y. Kozlovsky and M. M. Kozlov , “Membrane Fission: Model for Intermediate Structures,” Biophysical Journal 85, no. 1 (2003): 85–96. 12829467 10.1016/S0006-3495(03)74457-9 PMC1303068

[332] T. S. Gomez and D. D. Billadeau , “A FAM21‐Containing WASH Complex Regulates Retromer‐Dependent Sorting,” Developmental Cell 17, no. 5 (2009): 699–711. 19922874 10.1016/j.devcel.2009.09.009 PMC2803077

[333] D. Jia , T. S. Gomez , D. D. Billadeau , and M. K. Rosen , “Multiple Repeat Elements Within the FAM21 Tail Link the WASH Actin Regulatory Complex to the Retromer,” Molecular Biology of the Cell 23, no. 12 (2012): 2352–2361. 22513087 10.1091/mbc.E11-12-1059 PMC3374753

[334] M. N. Seaman , A. Gautreau , and D. D. Billadeau , “Retromer‐Mediated Endosomal Protein Sorting: All WASHed Up!,” Trends in Cell Biology 23, no. 11 (2013): 522–528. 23721880 10.1016/j.tcb.2013.04.010 PMC3924425

[335] E. Derivery , B. Lombard , D. Loew , and A. Gautreau , “The Wave Complex Is Intrinsically Inactive,” Cell Motility and the Cytoskeleton 66, no. 10 (2009): 777–790. 19206172 10.1002/cm.20342

[336] D. Frisby , A. B. Murakonda , B. Ashraf , et al., “Endosomal Actin Branching, Fission, and Receptor Recycling Require FCHSD2 Recruitment by MICAL‐L1,” Molecular Biology of the Cell 35, no. 11 (2024): ar144. 39382837 10.1091/mbc.E24-07-0324 PMC11617095

[337] M. A. Puthenveedu , B. Lauffer , P. Temkin , et al., “Sequence‐Dependent Sorting of Recycling Proteins by Actin‐Stabilized Endosomal Microdomains,” Cell 143, no. 5 (2010): 761–773. 21111236 10.1016/j.cell.2010.10.003 PMC3058345

[338] J. F. Striepen and G. K. Voeltz , “Coronin 1C Restricts Endosomal Branched Actin to Organize ER Contact and Endosome Fission,” Journal of Cell Biology 221, no. 8 (2022): e202110089. 35802042 10.1083/jcb.202110089 PMC9274145

[339] M. J. Hoyer , P. J. Chitwood , C. C. Ebmeier , et al., “A Novel Class of ER Membrane Proteins Regulates ER‐Associated Endosome Fission,” Cell 175, no. 1 (2018): 254–265.e14. 10.1016/j.cell.2018.08.030 PMC6195207 30220460

[340] K. Dhawan , N. Naslavsky , and S. Caplan , “Coronin2A Links Actin‐Based Endosomal Processes to the EHD1 Fission Machinery,” Molecular Biology of the Cell 33, no. 12 (2022): ar107. 35921168 10.1091/mbc.E21-12-0624 PMC9635295

[341] S. N. Duleh and M. D. Welch , “WASH and the Arp2/3 Complex Regulate Endosome Shape and Trafficking,” Cytoskeleton 67, no. 3 (2010): 193–206. 20175130 10.1002/cm.20437 PMC2887680

[342] N. Naslavsky and S. Caplan , “Advances and Challenges in Understanding Endosomal Sorting and Fission,” FEBS Journal 290, no. 17 (2022): 4187–4195. 36413090 10.1111/febs.16687 PMC10200825

